# Bone marrow stromal cells from **β**-thalassemia patients have impaired hematopoietic supportive capacity

**DOI:** 10.1172/JCI123191

**Published:** 2019-02-25

**Authors:** Stefania Crippa, Valeria Rossella, Annamaria Aprile, Laura Silvestri, Silvia Rivis, Samantha Scaramuzza, Stefania Pirroni, Maria Antonietta Avanzini, Luca Basso-Ricci, Raisa Jofra Hernandez, Marco Zecca, Sarah Marktel, Fabio Ciceri, Alessandro Aiuti, Giuliana Ferrari, Maria Ester Bernardo

**Affiliations:** 1 San Raffaele Telethon Institute for Gene Therapy (SR-TIGET), and; 2 Regulation of Iron Metabolism Unit, Division of Genetics and Cell Biology, IRCCS San Raffaele Scientific Institute, Milan, Italy.; 3 Oncoematologia Pediatrica, Fondazione IRCCS Policlinico “San Matteo”, Pavia, Italy.; 4 Hematology and Bone Marrow Transplantation Unit, IRCCS San Raffaele Scientific Institute, Milan, Italy.; 5 Vita-Salute San Raffaele University, Milan, Italy.; 6 Pediatric Immunohematology and Bone Marrow Transplantation Unit, IRCCS San Raffaele Scientific Institute, Milan, Italy.

**Keywords:** Stem cells, Transplantation, Bone marrow, Cell stress, Stem cell transplantation

## Abstract

**BACKGROUND.** The human bone marrow (BM) niche contains a population of mesenchymal stromal cells (MSCs) that provide physical support and regulate hematopoietic stem cell (HSC) homeostasis. β-Thalassemia (BT) is a hereditary disorder characterized by altered hemoglobin beta-chain synthesis amenable to allogeneic HSC transplantation and HSC gene therapy. Iron overload (IO) is a common complication in BT patients affecting several organs. However, data on the BM stromal compartment are scarce.

**METHODS.** MSCs were isolated and characterized from BM aspirates of healthy donors (HDs) and BT patients. The state of IO was assessed and correlated with the presence of primitive MSCs in vitro and in vivo. Hematopoietic supportive capacity of MSCs was evaluated by transwell migration assay and 2D coculture of MSCs with human CD34^^+^^ HSCs. In vivo, the ability of MSCs to facilitate HSC engraftment was tested in a xenogenic transplant model, whereas the capacity to sustain human hematopoiesis was evaluated in humanized ossicle models.

**RESULTS.** We report that, despite iron chelation, BT BM contains high levels of iron and ferritin, indicative of iron accumulation in the BM niche. We found a pauperization of the most primitive MSC pool caused by increased ROS production in vitro which impaired MSC stemness properties. We confirmed a reduced frequency of primitive MSCs in vivo in BT patients. We also discovered a weakened antioxidative response and diminished expression of BM niche–associated genes in BT-MSCs. This caused a functional impairment in MSC hematopoietic supportive capacity in vitro and in cotransplantation models. In addition, BT-MSCs failed to form a proper BM niche in humanized ossicle models.

**CONCLUSION.** Our results suggest an impairment in the mesenchymal compartment of BT BM niche and highlight the need for novel strategies to target the niche to reduce IO and oxidative stress before transplantation.

**FUNDING.** This work was supported by the SR-TIGET Core grant from Fondazione Telethon and by Ricerca Corrente.

## Introduction

β-Thalassemia (BT) is one of the most common inherited monogenic disorders in the world, with an overall carrier rate of 1.5% of the world population, and 60,000 BT symptomatic patients born per year (1, 2). Mutations in the gene encoding for the human *β-globin* gene result in a reduction in or absence of the beta-globin chains, leading to the accumulation of unstable α-hemoglobin, which is responsible for the pathophysiology of the disorder (3–5). Conventional treatment of BT relies on chronic and regular blood transfusions in association with iron-chelation therapy (6, 7). However, complications caused by iron accumulation and hepcidin dysregulation due to expanded ineffective erythropoiesis still affect quality of life and represent a cause of death (8–12). The only curative treatment for BT patients is receipt of an allogeneic hematopoietic stem cell (HSC) transplant from a compatible donor, which leaves half of the patients without a definitive cure due to unavailability of matched donors (13–19). More recently, gene therapy (GT) with autologous HSCs modified ex vivo to restore β-globin expression has shown promising results in preclinical animal models and in clinical trials for BT (20–25), offering the possibility for a definitive cure to a large number of BT patients who lack a matched donor. In the transplant context, the presence of a functional bone marrow (BM) microenvironment capable of sustaining HSC engraftment, expansion, and differentiation is a fundamental requisite for a successful outcome (26).

The human BM niche includes several nonhematopoietic cells. Among these are mesenchymal stromal cells (MSCs), which offer physical support to HSCs and tightly control their fate (27–32). Different subtypes of MSCs interact with HSCs in specific regions of the BM niche, including CD271^+^ and CD146^+^ MSCs that have been described as primitive MSCs associated with long-term HSCs (33–36). Despite MSCs only accounting for approximately 0.001%–0.01% of mononuclear cells (MNCs) in human BM (37), they can be efficiently isolated from BM-MNCs and expanded in vitro thanks to their ability to adhere to plastic. Ex vivo–expanded MSCs are defined based on their spindle fibroblast-like morphology, expression of specific surface markers, and capability to differentiate into mesodermal lineages (38–42). Apart from their stem/stromal features, MSCs are characterized by both antiinflammatory and proinflammatory properties (43–45). Because of these properties, MSCs have been employed in clinical settings of HSC transplantation to facilitate HSC engraftment and rescue patients with steroid-resistant acute graft-versus-host disease (46–51).

We hypothesize that in BT patients several stress signals, including oxidative stress, inflammation, and hypoxia derived from ineffective erythropoiesis, may alter the BM niche. Moreover, a negative impact of the altered microenvironment on HSC function has been shown in a mouse model of BT and in conditions of iron overload (IO) (11, 52–54). Whether the BM microenvironment of BT patients is impaired, particularly at the cellular and molecular levels, and what mechanisms are involved in this injury, have not been clearly defined. In this work, we have characterized the biological and functional properties of MSCs obtained from BM of BT patients, and analyzed the role of IO on the hematopoietic supportive capacity of the BT mesenchymal compartment in vitro and in vivo.

## Results

### Isolation and characterization of MSCs from BT patients and healthy donor controls (HDs).

**Figure 1:**
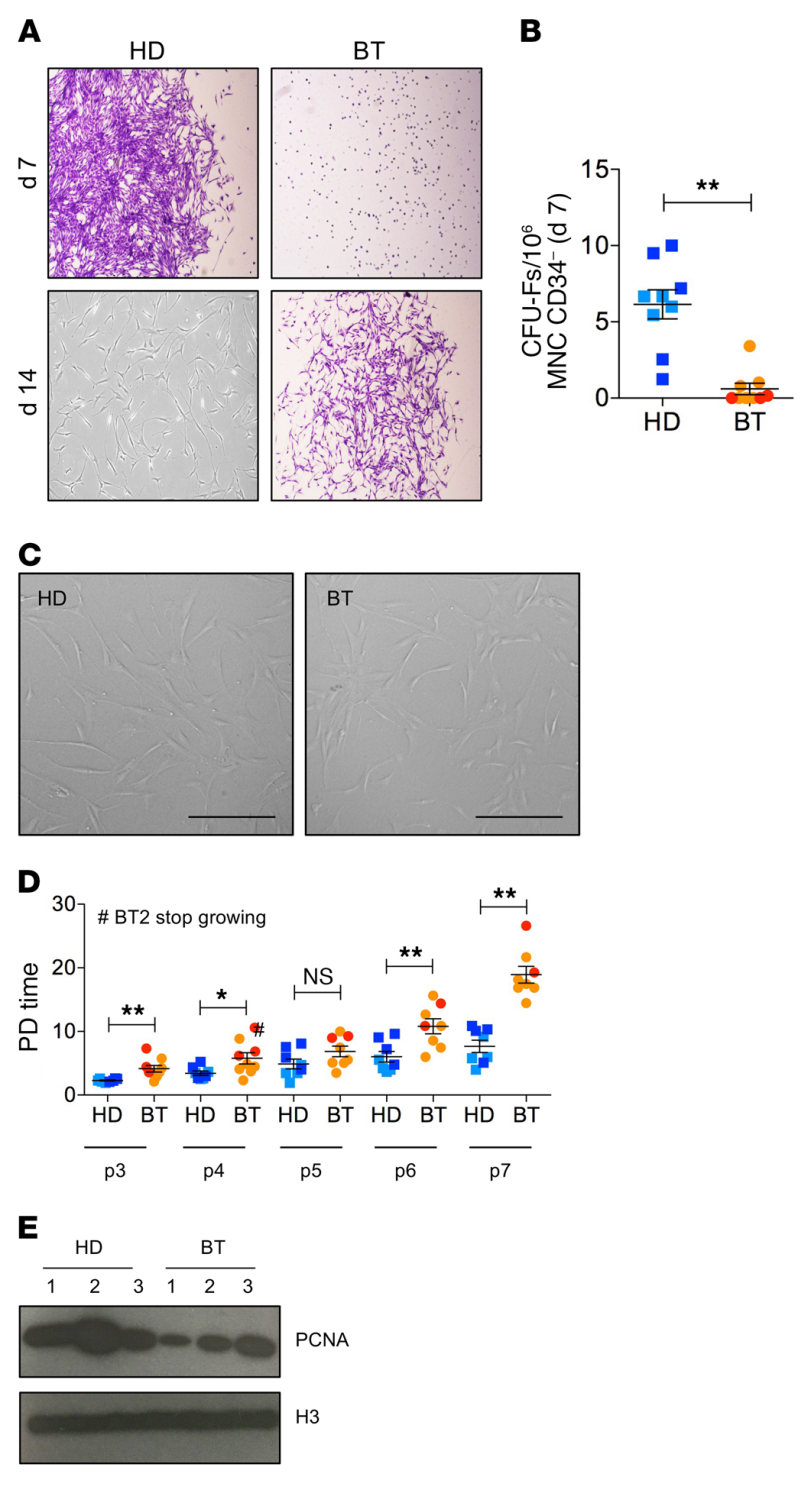
Clonogenic capacity, morphology, and proliferation ability of HD- and BT-MSCs. (**A**) Representative images of fibroblast colony-forming units (CFU-Fs) stained with crystal violet at 7 and 14 days after plating CD34^–^ MNCs purified from BM samples of BT patients and HD controls. Original magnification, ×4. (**B**) CFU assay for HD-MSCs (*n* = 9) and BT-MSCs (*n* = 8). Results are expressed as number of colonies per 10^6^ CD34^–^ MNCs. Error bars show mean ± SEM. (**C**) Representative examples of in vitro–expanded MSC morphology obtained from HD and BT samples. Scale bars: 200 μM. (**D**) Population doubling (PD) time of HD-MSCs (*n* = 8) and BT-MSCs (*n* = 8) calculated from passage 3 (p3) to p7. Error bars show mean ± SEM. #Indicates the BT sample, which stopped growing at p4. (**E**) Western blot analysis of PCNA expression on nuclear protein extracts isolated from 3 different HD- and BT-MSCs. H3 antibody was used for normalization. In all panels, each square represents one HD sample (blue: >18 years; light blue: <18 years). Each circle represents one BT sample (red: >18 years; orange: <18 years). *P* values were determined by Student’s *t* test (*P < 0.05; **P < 0.001).

MSCs were isolated from BM aspirates of BT patients according to standard protocols (55). Similarly, MSCs were isolated from age-matched HD BM samples. HD-MSCs appeared as clones of fibroblast-like cells (CFU-Fs) starting from 5 to 7 days after plating. On the contrary, we observed a delay in the outgrowing and a significantly reduced number of CFU-Fs for BT samples (*P* < 0.0001) (Figure 1, A and B), highlighting an impaired clonogenic capacity of BT-MSCs. Despite a similar fibroblast-like morphology in culture (Figure 1C), BT-MSCs proliferated significantly slower than the HD counterparts (Figure 1D), underwent premature replicative senescence (median passage: HD = P10; BT = P7), and expressed a lower level of proliferating cell nuclear antigen (PCNA) (Figure 1E).

**Figure 2:**
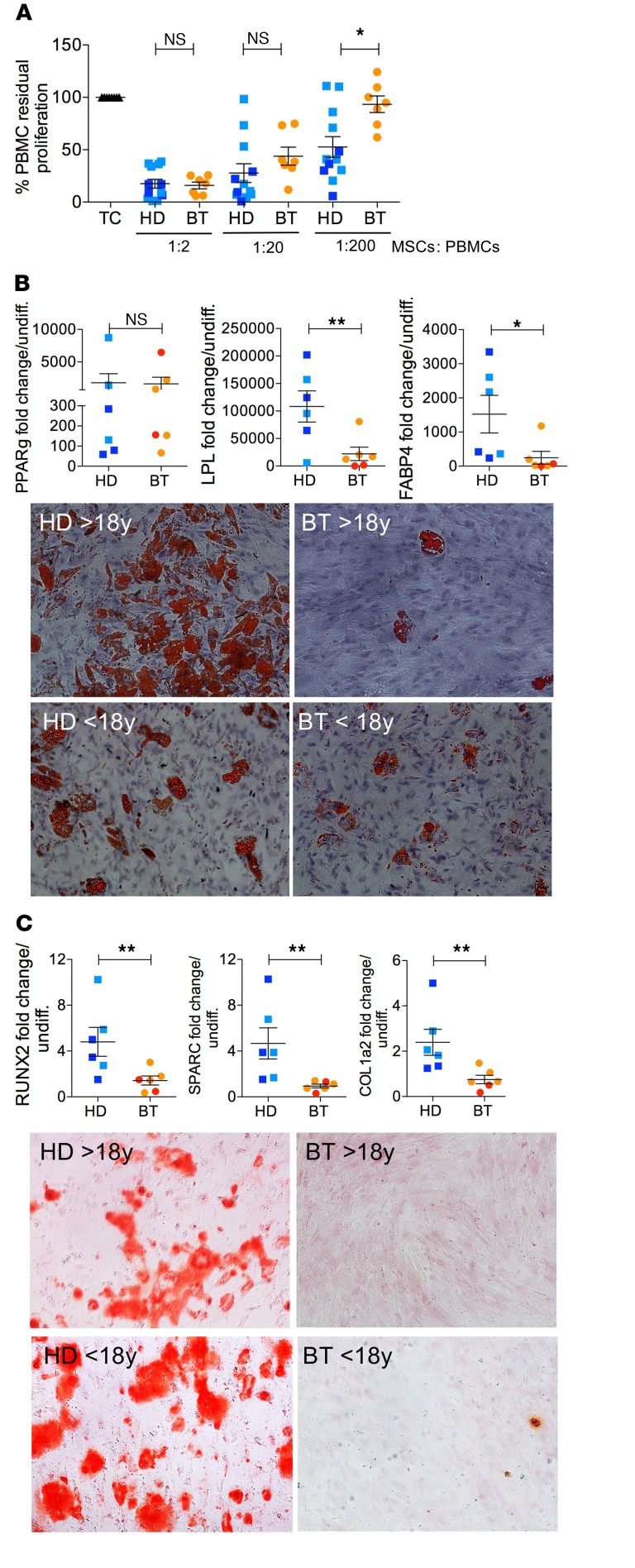
Functional characterization of HD- and BT-MSCs. (**A**) The in vitro immunomodulatory effect of HD- and BT-MSCs on human healthy donor PBMCs in an allogenic setting. The graph shows the percentage of residual proliferation of PHA-stimulated PBMCs either in absence (TC) or presence of HD- or BT-MSCs at different ratios (1:2, 1:20, and 1:200), calculated by measuring 3H-thymidine incorporation after 72 hours of coculture. We referred to PBMC proliferation in the absence of MSCs as 100%, and this percentage was used to normalize PBMC proliferation in the presence of MSCs. Each error bar show mean ± SEM (HD: *n* = 12; BT: *n* = 7). (**B**) Expression of early (*PPARg*) and late (*LPL*, *FABP4*) adipogenic genes in HD- and BT-MSCs induced to differentiate in proper adipogenic medium for 21 days (upper panel). Results are expressed as fold change relative to undifferentiated MSCs (HD: *n* = 6; BT: *n* = 6). Each error bar shows mean ± SEM. Representative images of differentiated HD- and BT-MSCs stained with Oil Red O (lower panel, original magnification ×10). (**C**) Real-time PCR for early (*RUNX2*) and late (*SPARC*, *COL1A2*) osteogenic gene expression in HD- and BT-MSCs induced to differentiate in proper osteogenic medium for 21 days (upper panel). Each error bar shows mean ± SEM of gene expression as fold change relative to undifferentiated MSCs (HD: *n* = 6; BT: *n* = 6). Representative images of differentiated HD- and BT-MSCs stained with Alizarin Red S (lower panel, original magnification ×10). In all panels, each square represents one HD sample (blue: >18 years; light blue: <18 years). Each circle represents one BT sample (red: >18 years; orange: <18 years). *P* values were determined by Student’s *t* test (*P < 0.05; **P < 0.001).

**Figure 3:**
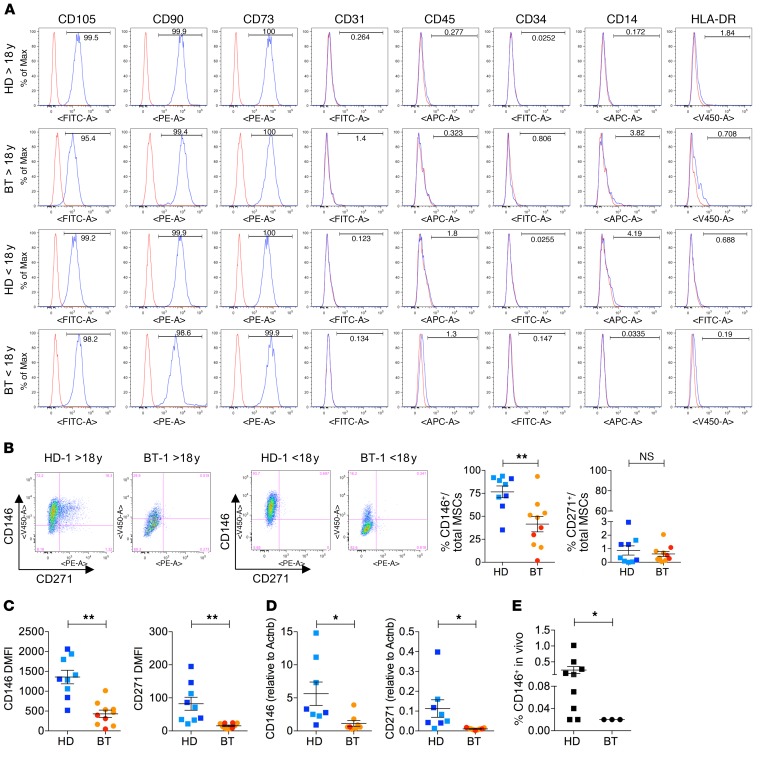
Immunophenotypic characterization of HD- and BT-MSCs. (**A**) Representative images of flow cytometry analysis of in vitro–expanded HD- and BT-MSCs. Canonical MSC markers: CD105, CD90, CD73. Endothelial marker: CD31. Hematopoietic markers: CD45, CD34, CD14. MHC class II marker: HLA-DR. (**B**) Representative images of CD146 and CD271 expression analysis by flow cytometry in adult (>18 years) and pediatric (<18 years) HD- and BT-MSCs (left panel). Frequencies of CD146^+^ and CD271^+^ cells are expressed as percentages of total MSCs (right panel). Each error bar shows mean ± SEM (HD: *n* = 9; BT: *n* = 10). (**C**) Level of CD146 and CD271 expression represented as dMFI relative to unstained controls. Each error bar shows mean ± SEM (HD: *n* = 9; BT: *n* = 10). (**D**) Real-time qPCR analysis for CD146 and CD271 expression in HD-MSCs (*n* = 8) and BT-MSCs (*n* = 9). Results are expressed as ΔΔCT. Each error bar shows mean ± SEM. (**E**) Frequency of CD146^+^ expressed as a percentage of total MNCs isolated from HD (*n* = 6) and BT (*n* = 3) BM aspirates. Each error bar shows mean ± SEM. In all panels, each square represents one HD sample (blue: >18 years; light blue: <18 years). Each circle represents one BT sample (red: >18 years; orange: <18 years). *P* values were determined by Student’s *t* test (*P < 0.05; **P < 0.001).

We further characterized the immunoregulatory properties, differentiation capacities, and immunophenotypes of BT- and HD-MSCs. To evaluate the immunoregulatory activity of MSCs on peripheral blood mononuclear cells (PBMCs), allogeneic phytohemagglutin-stimulated (PHA-stimulated) PBMCs were cultured in the presence or absence of HD- or BT-MSCs at different MSC-to-PBMC ratios. BT-MSCs were effective in inhibiting PBMC proliferation to the same extent as HD-MSCs when plated at a high MSC-to-PBMC ratio. On the contrary, we observed a more robust antiproliferative effect of HD-MSCs on PHA-stimulated PBMCs in the presence of a low number of MSCs (ratio 1:200) (Figure 2A). Next, we induced adipogenic and osteogenic differentiation and analyzed the induction of lineage determination and tissue-specific genes in HD- and BT-MSCs. BT-MSCs failed to efficiently differentiate into adipocytes and to form bone. In fact, whereas *PPARg*, a master regulator of adipogenesis, was similarly activated in HD- and BT-MSCs (Figure 2B), the expression of *LPL* and *FABP4* was significantly impaired in BT-MSCs and correlated with reduced lipid droplet formation (Figure 2B). When we analyzed the capability of MSCs to form bone, we found that *RUNX2* was efficiently activated in HD-MSCs, but not in BT-MSCs. As a consequence, *SPARC* and *COL1A2* were downregulated in BT-MSCs, which failed to form mineralized bone (Figure 2C). Immunophenotypic analysis showed that ex vivo–expanded BT- and HD-MSCs expressed the canonical MSC markers CD105, CD90, and CD73. They lacked the expression of endothelial (CD31), hematopoietic (CD45, CD34), and monocyte/macrophage (CD14) markers, and did not express HLA-DR (Figure 3A). We also evaluated the expression of CD146 and CD271, recently described as markers of primitive, highly clonogenic, self-renewing, and multipotent MSCs interacting with HSCs in specific BM regions (33–36, 56, 57). A robust reduction of CD146^+^ cell frequency and expression was observed in BT-MSCs compared with HDs (Figure 3, B–D). The frequency of CD271^+^ cells was extremely low compared with CD146^+^ cells, according to reported data that indicated a rapid downregulation of CD271 in culture (Figure 3B) (58). However, we were able to detect a decreased expression of CD271 in BT-MSCs (Figure 3, C and D). In conclusion, despite a canonical morphology and immunophenotypic profile, BT-MSCs showed an altered clonogenic capacity, a lower proliferation rate, and an inefficient differentiation capacity compared with HD controls. These alterations correlated with a robust pauperization of the most primitive stromal cell pool in vitro, as suggested by decreased expression of CD146 and CD271 markers. A reduced frequency of CD146^+^ cells was found in vivo when sorting CD45^–^, CD105^+^, and CD146^+^ cells from BM aspirates of BT patients, demonstrating that the alteration found in the primitive MSC compartment was not only an in vitro artifact (Figure 3E).

### Iron uptake and storage in MSCs exposed to IO.

**Figure 4:**
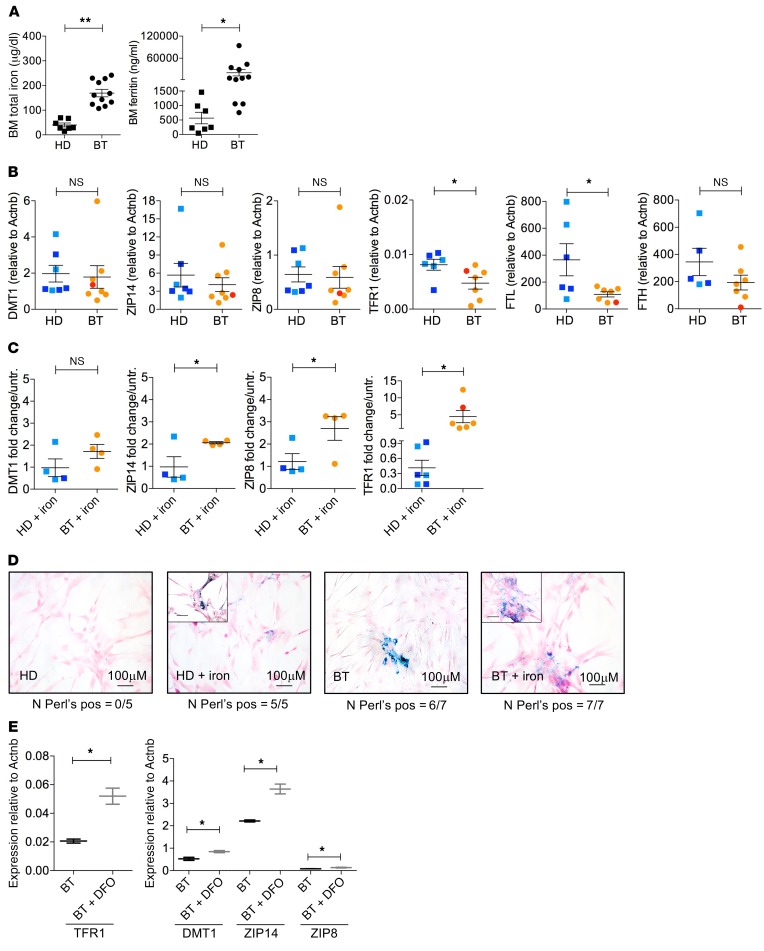
Iron metabolism in HD- and BT-MSCs. (**A**) Quantification of total iron (μg/dl) and ferritin level (ng/ml) in the plasma of HD (*n* = 7) and BT (*n* = 11) BM aspirates. Each error bar shows mean ± SEM. (**B**) Expression of free iron transporter (*DMT1*, *ZIP14*, *ZIP8*), transferrin (*TFR1*), and ferritin (*FTL*, *FTH*) genes at the basal level in HD-MSCs (*n* = 7) and BT-MSCs (*n* = 8). Results are expressed as ΔΔCT. Each error bar shows mean ± SEM. (**C**) Expression of free iron transporter (*DMT1*, *ZIP14*, *ZIP8*), transferrin (*TFR1*), and ferritin (*FTL*, *FTH*) genes in HD-MSCs (*n* = 6) and BT-MSCs (*n* = 6) exposed to 40 μM ferric ammonium citrate (+ iron) for 5 days. Results are expressed as fold change relative to untreated MSCs. Each error bar shows mean ± SEM. In all panels, each square represents one HD sample (blue: >18 years; light blue: <18 years). Each circle represents one BT sample (red: >18 years; orange: <18 years). *P* values were determined by Student’s *t* test (*P < 0.05; **P < 0.001). (**D**) Representative images of iron deposition assessment in HD- and BT-MSCs at the basal level and after 5 days of 40 μM iron treatment (+ iron), using Perl’s staining. Number of samples positive for iron deposits are indicated below each panel. (**E**) qPCR expression analysis of free iron transporter (*DMT1*, *ZIP14*, *ZIP8*) and transferrin (*TFR1*) genes in untreated BT-MSCs (black) and in BT-MSCs treated with 100 μM DFO (+ DFO) for 24 hours (gray). Data are mean ± SEM. Experiments were performed in triplicate; *n* = 3. *P* values were determined by Student’s *t* test (*P < 0.05).

Considering that iron is a potent source of ROS and increased levels of ROS are known to promote quiescence exit and stem cell activation in several biological systems (59, 60), we investigated the IO state of the BM niche by measuring the concentrations of total iron and ferritin in the plasma of BM aspirates obtained from BT patients (*n* = 11) and controls (*n* = 7). Despite the regular use of iron chelation in patients, we observed remarkably higher levels of total iron and ferritin in BT BM samples compared with controls (Figure 4A), with ferritin levels strikingly more elevated than the corresponding patient’s peripheral blood values (mean total iron 171.77 μg/dl, range 69–236 μg/dl; mean ferritin 1617.6 ng/ml, range 181–3226 ng/ml; *P* = 0.05). This indicates a local state of IO, regardless of treatment with iron chelators, which may generate oxidative stress, altering the functional properties of MSCs (11, 52, 61–63). We then studied whether MSCs were able to uptake and store iron excess, by analyzing the expression of iron transporter and ferritin genes. BT- and HD-MSCs expressed iron transporters (*DMT1*, *ZIP14*, *ZIP18*) at similar levels, whereas the expression of *TFR1* was reduced in BT-MSCs (Figure 4B). At the basal level, *FTL* and *FTH* genes were highly expressed in MSCs, suggesting that MSCs may act as iron storage cells in the case of IO. The *FTL* gene was significantly more expressed in HD-MSCs compared with BT-MSCs, with a similar trend for the *FTH* gene (Figure 4B). Next, we evaluated the capacity of MSCs to uptake and store iron by treating cells with increasing doses of iron in culture (Figure 4C and Supplemental Figure 1A; supplemental material available online with this article; https://doi.org/10.1172/JCI123191DS1), provided as ferric ammonium citrate (FAC) (64–66). Exposure to FAC reproduces in vitro non–transferrin-bound iron, which exceeds the buffering capacity of transferrin in BT patients (67). In particular, we used 40 μM FAC, which mimics the amount of iron measured in the BM plasma of BT patients (average value of total iron in BT BM plasma: 169.45 μg/dl, corresponding to 30.72 μM). Iron transporters (*ZIP14*, *ZIP18*) were induced in BT-MSCs more efficiently than in HD controls. A similar trend was observed for *DMT1* expression (Figure 4C). As expected, *TFR1* was downregulated in HD-MSCs exposed to iron (63, 68), whereas BT-MSCs upregulated the expression of *TFR1*, indicating an impairment in the iron-sensing machinery in vitro under IO conditions (Figure 4C) (63, 68). In line with this, we observed a blunted upregulation of ferritin in BT-MSCs exposed to iron compared with controls (Supplemental Figure 1B). Perl’s staining confirmed the ability of MSCs to uptake iron after in vitro exposure to a high concentration of iron (Figure 4D). Importantly, we observed Perl’s-positive cells in 6 of 7 BT-MSCs also at the basal level (i.e., before in vitro iron treatment) at early passages (P1–P2) in standard conditions. The Perl’s-positive cells were never detected in control samples (Figure 4D). We reasoned that BT-MSCs may uptake and accumulate iron in vivo and retain some of this iron. In addition, we checked the expression of iron transporter genes in MSCs exposed to different doses of iron, considering iron fluctuation in the blood stream. We observed an inappropriate regulation of iron transporters in BT-MSCs treated with 5, 10, and 20 μM iron for 5 days (Supplemental Figure 1A). Finally, we treated HD- and BT-MSCs with 40 μM iron for a prolonged period (21 days) to mimic in vivo chronic exposure. HD- and BT-MSCs accumulated intracellular iron, as shown by Perl’s staining, and acquired a flat and enlarged morphology typical of senescent cells (Supplemental Figure 1C). We couldn’t extend iron treatment for longer time points because cells died. We confirmed that iron treatment activates a senescent program in MSCs by testing the expression of *CDKN2A*, which was upregulated in HD- and BT-MSCs after exposure with a trend for a more robust upregulation in BT-MSCs (Supplemental Figure 1D). We concluded that, like other BM cell types (69, 70), BM-MSCs are able to uptake iron excess, possibly to protect HSC homeostasis and function (71). However, we observed a dysregulation of the iron-sensing machinery in BT-MSCs, possibly due to prolonged iron exposure in vivo, which caused an excessive iron accumulation in vitro (Figure 4, A–D and Supplemental Figure 1A).

When we exposed BT-MSCs to an iron-chelating agent (deferoxamine [DFO] 100 μM for 24 hours), we observed a significant upregulation of *TFR1* and *ZIP14* in BT-MSCs treated with DFO compared with untreated cells (Figure 4E). Similarly, a trend of upregulated expression of *DMT1* and *ZIP8* was observed in DFO-treated cells (Figure 4E). We further evaluate the effect of DFO treatment on BT-MSCs in the presence of iron. The expression of *TFR1* and DMT1 was inhibited by iron in DFO-treated cells (Supplemental Figure 2), indicating a proper function of the iron regulatory axis in acute treatment.

### Oxidative stress reduces the frequency of primitive MSCs.

**Figure 5:**
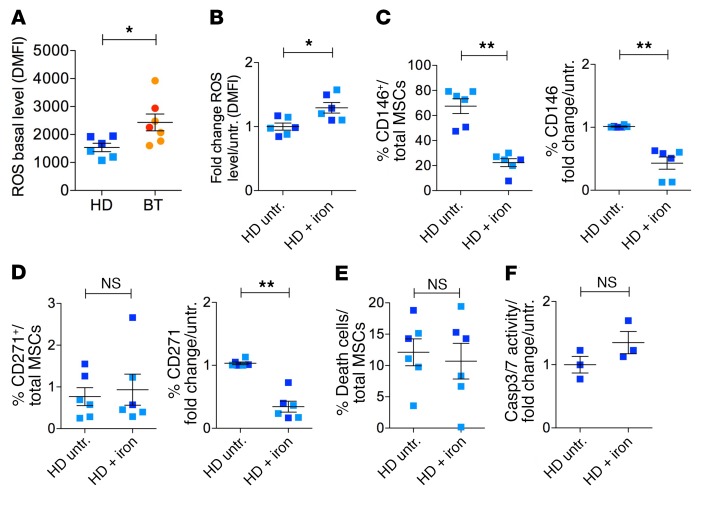
Reduced expression of primitive MSC markers inversely correlates with ROS levels. (**A**) Flow cytometry analysis of ROS levels in HD-MSCs (*n* = 6) and BT-MSCs (*n* = 7) using CellROX assay. Results are expressed as dMFI relative to unstained control. (**B**) Measurement of ROS levels in HD-MSCs exposed to 40 μM iron for 5 days (*n* = 6) compared with untreated samples (*n* = 6). Each error bar shows mean ± SEM. (**C**) Frequency of CD146^+^ expressed as percentage of total cells in untreated (*n* = 6) and iron-exposed (*n* = 6) HD-MSCs (left panel). CD146 expression after iron treatment in HD-MSCs (*n* = 6) by RT-qPCR. Results are expressed as fold change relative to untreated samples (*n* = 6) (right panel). (**D**) Frequency of CD271^+^ in untreated (*n* = 6) and iron-exposed (*n* = 6) HD-MSCs (left panel). CD271 expression after iron treatment in HD-MSCs (*n* = 6) by RT-qPCR. Results are expressed as fold change relative to untreated samples (*n* = 6) (right panel). (**E**) Percentage of trypan blue–positive cells in HD-MSCs before (*n* = 6) and after (*n* = 6) iron treatment. (**F**) Analysis of caspase 3/7 activity in untreated and iron-treated HD-MSCs. In all panels, error bars show mean ± SEM. Each square represents one HD sample (blue: >18 years; light blue: <18 years). Each circle represents one BT sample (red: >18 years; orange: <18 years). *P* values were determined by Student’s *t* test (*P < 0.05; **P < 0.001). HD untr.: HD untreated; HD + iron: iron-treated HD.

**Figure 6:**
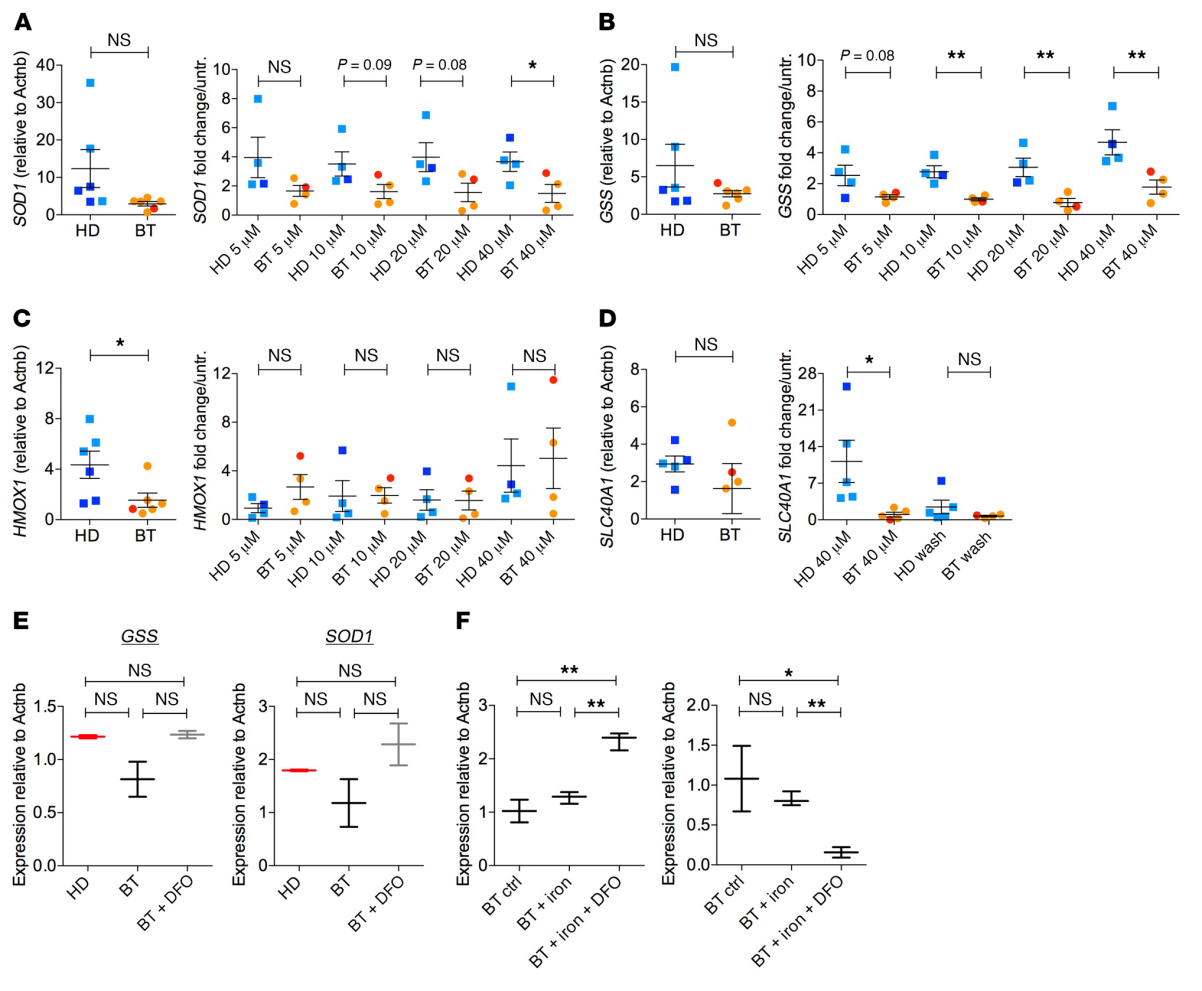
Impaired antioxidant stress response in BT-MSCs. (**A**–**C**) Expression of superoxide dismutase 1 (*SOD1*), glutathione synthetase (*GSS*), and heme oxygenase 1 (*HMOX1*) genes in HD-MSCs (*n* = 6) and BT-MSCs (*n* = 6). Results are expressed as ΔΔCT (left panel). Analysis of *SOD1*, *GSS*, and *HMOX1* expression in HD-MSCs (*n* = 6) and BT-MSCs (*n* = 6) after treatment with increasing doses of iron (5, 10, 20, 40 μM). Results are expressed as fold change relative to untreated controls (right panel). Error bars show mean ± SEM. (**D**) Expression of ferroportin (*SLC40A1*) in HD- and BT-MSCs at the basal level (left panel), exposed to iron (40 μM), and cultured in DMEM after iron exposure (wash) (right panel). Results are expressed as ΔΔCT (left panel) or as fold change relative to untreated controls (right panel). Error bars show mean ± SEM. (HD: *n* = 5; BT: *n* = 4). In all panels, each square represents one HD sample (blue: >18 years; light blue: <18 years). Each circle represents one BT sample (red: >18 years; orange: <18 years). *P* values were determined by Student’s *t* test (*P < 0.05; **P < 0.001). (**E**) Analysis of *GSS* and *SOD1* expression in untreated HD controls (red), untreated BT-MSCs (black), and BT-MSCs treated with 100 μM DFO for 24 hours (BT + DFO, gray). (**F**) qPCR analysis for *GSS* and *SOD1* expression in untreated BT-MSCs, BT-MSCs exposed to iron (40 μM), and BT-MSCs exposed to iron and treated with 100 μM DFO for 24 hours (BT + iron + DFO). Data are mean ± SEM. Experiments were performed in triplicate; *n* = 3. *P* values were determined by Student’s *t* test (*P < 0.05; **P < 0.001).

We hypothesized that prolonged iron exposure could generate high levels of ROS, inducing BT-MSCs to exit the primitive state. We measured ROS levels by flow cytometry and found significantly higher levels of ROS in BT-MSCs compared with HD-MSCs (Figure 5A), which inversely correlated with CD146 and CD271 expression (Figure 3, B–D). In addition, we observed that iron exposure in vitro significantly increased ROS levels (Figure 5B) and reduced CD146 frequency and expression in iron-treated HD-MSCs (Figure 5C). The frequency of CD271^+^ cells was not altered after iron treatment in HD-MSCs, whereas CD271 expression was significantly reduced (Figure 5D). The content of dead and apoptotic cells was similar in untreated and iron-exposed HD-MSCs, demonstrating that reduction of primitive MSCs was mainly caused by ROS-induced primitive state exit (Figure 5, E and F). We further investigated whether the exit from primitive state was linked to the activation of a senescent program in HD- and BT-MSCs at the basal level and after iron treatment. We found a trend for an increased expression of senescent-associated secretory phenotype (SASP) factors (IL1b, IL1a, and MIP-1a) in BT-MSCs compared with controls (Supplemental Figure 3A). This may indicate that BT-MSCs are exhausted in attempts to counteract IO in vivo. Moreover, we showed a robust stabilization of p53 after 5 days of iron treatment in BT-MSCs (Supplemental Figure 3B). We concluded that ROS generation after iron exposure correlated with a significant reduction of primitive MSCs in vitro. This mechanism may reflect a molecular alteration causing the reduction of CD146^+^ cells in vivo (Figure 3E). Importantly, ROS levels were increased in BT-MSCs compared with controls at different passages in culture despite BT-MSCs possibly having released iron deposits during ex vivo expansion, given their known ability to uptake and release molecules (72–74). To explain this finding, we investigated the efficiency of antioxidant response in BT- and HD-MSCs at the basal level and in response to different concentrations of iron. We found that the expression of superoxide dismutase 1 (*SOD1*) and glutathione synthetase (*GSS*) was reduced, although not significantly, in BT-MSCs compared with HD-MSCs, whereas a significantly lower expression of heme-oxygenase 1 (*HMOX1*) was observed in BT-MSCs, indicating a reduced stress defense (Figure 6, A–C). The inability to properly react against an oxidative stress was already present in BT-MSCs treated with low doses of iron and was more evident with dose increase. In particular, BT-MSCs failed to robustly induce *SOD1*, especially at a high iron dose (40 μM), whereas the expression of *GSS* was reduced in BT-MSCs compared with HD controls starting from lower doses of iron and becoming significant at 10 μM iron (Figure 6, A and B). On the contrary, the expression level of *HMOX1* was similar in BT-MSCs and HD controls when treated with iron (Figure 6C).

Induction of ferroportin (*SLC40A1*) represents a protection mechanism against cytoplasmic free iron accumulation (75, 76). We analyzed the expression of *SLC40A1* in BT- and HD-MSCs at the basal level and after 5 days of iron exposure. While HD-MSCs robustly activated *SLC40A1* when exposed to iron, BT-MSCs failed to rapidly induce *SLC40A1*, indicating an impairment in the iron export mechanism. When iron-treated HD-MSCs were cultured in normal medium (i.e., without iron), the expression of *SLC40A1* returned to the basal level (Figure 6D). We concluded that the antioxidant response and the protection mechanisms against iron accumulation and oxidative stress were impaired in BT-MSCs, which failed to manage stress-induced ROS.

When we exposed BT-MSCs to DFO, we did not observe a significant improvement in the antioxidant capacity of DFO-treated BT-MSCs, but only a trend toward a better response (Figure 6E). When we treated BT-MSCs with iron and DFO, we observed a robust induction of *GSS*. On the contrary, *SOD1* expression was still dysregulated in DFO-treated cells (Figure 6F). A more robust effect could possibly be obtained by prolonged exposure to DFO, considering that BT-MSCs have been exposed for a long time to IO, which may have established an epigenetic memory in BT-MSCs, thus affecting their functionality. Finally, the use of an iron-chelating agent could represent an option to at least partly restore the iron metabolic response of BT-MSCs in vitro.

### Reduced hematopoietic supportive capacity of BT-MSCs in vitro and in vivo.

**Figure 7:**
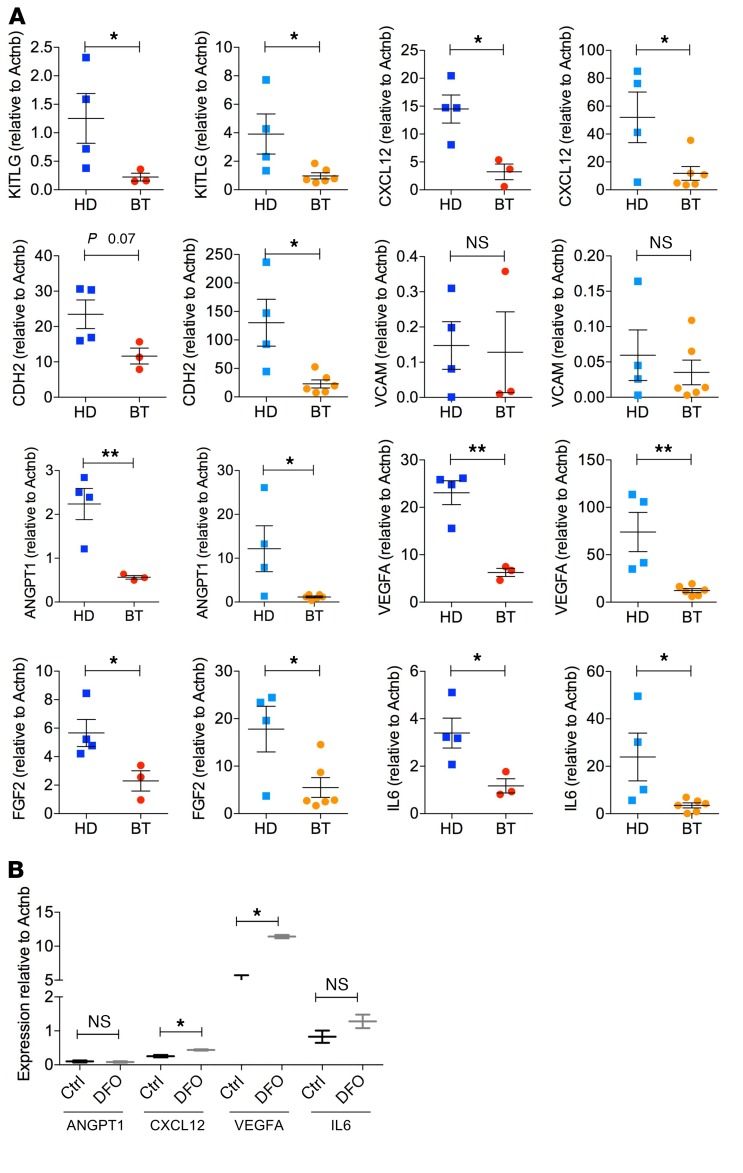
Reduced expression of BM niche–associated genes in BT-MSCs. (**A**–**C**) Expression analysis of BM niche–associated genes (*KITLG*, Kit ligand; *CXCL12*, C-C-C motif chemokine ligand 12; *CDH2*, cadherin 2; *VCAM1*, vascular cell adhesion molecule 1; *ANGPT1*, angiopoietin 1; *VEGFA*, vascular endothelial growth factor A; *FGF2*, fibroblast growth factor 2; *IL6*, interleukin 6) in adult and pediatric HD- and BT-MSCs. In all panels, each square represents one HD sample (blue: >18 years; light blue: <18 years). Each circle represents one BT sample (red: >18 years; orange: <18 years). Results are expressed as ΔΔCT (left panel). Each error bar shows mean ± SEM (adult HD: *n* = 4; adult BT: *n* = 3; pediatric HD: *n* = 4; pediatric BT: *n* = 6). (**B**) qPCR analysis of *ANGPT1*, *CXCL12*, *VEGFA*, and *IL6* expression in untreated BT-MSCs (black) and DFO-treated cells (+DFO, gray). Data are mean ± SEM. Experiments were performed in triplicate; *n* = 3. *P* values were determined by Student’s *t* test (*P < 0.05; **P < 0.001).

Considering the key role of MSCs in controlling HSC homeostasis and function, we investigated whether the hematopoietic supportive role of MSCs was preserved in BT samples despite the alterations in the primitive MSC compartment and increased ROS levels. Figure 7, A and B, shows our analysis of the expression of *KITLG* and *CXCL12,* which are critical for HSC engraftment, retention, survival, and proliferation (77, 78). *KITLG* and CXCL12 expression were significantly downregulated in BT-MSCs compared with HD controls, especially in MSCs isolated from adult BT patients. Several adhesion molecules are involved in the retention of HSCs within the BM and favor the lodgment of transplanted HSCs (79, 80). BT-MSCs expressed *CDH2* at a lower level compared with controls, whereas *VCAM* was similarly expressed by BT- and HD-MSCs. *ANGPT1* and *VEGFA*, which regulate HSPC quiescence (81), were also markedly downregulated in BT-MSCs. In addition, in BT-MSCs we observed a substantial reduction of *IL6* and *FGF2*, known to have a paracrine proliferative effect on MSCs and a supportive role for HSPC expansion (82, 83). We concluded that the expression of several BM niche–associated genes was reduced in BT patients. We speculated that prolonged iron exposure in vivo may induce an epigenetic remodeling of the BT-MSC gene expression profile, which could affect the expression of a broad set of genes, altering MSC functional properties. Indeed, several chromatin remodeling proteins and histone demethylases are iron responsive (84–86), including a large family of lysine-specific demethylases (KDMs), which are expressed in MSCs (Supplemental Figure 4A). A robust reduction of H3K9 and H3K36 methylation in BT-MSCs may highlight a different epigenetic state of cells exposed to iron (Supplemental Figure 4B). It’s noteworthy that 4 different published ChIP sequencing data sets showed that several BM niche–associated genes are specifically subjected to H3K36 methylation (Supplemental Figure 5).

We checked the expression of the major hematopoietic supportive factors that were found dysregulated in BT-MSCs after DFO exposure. Notably, we observed that the expression of *CXCL12* and *VEGFA* was significantly induced by DFO treatment. A trend of upregulated expression was also observed for *ANGPT1* and *IL6* after DFO exposure (Figure 7B).

**Figure 8:**
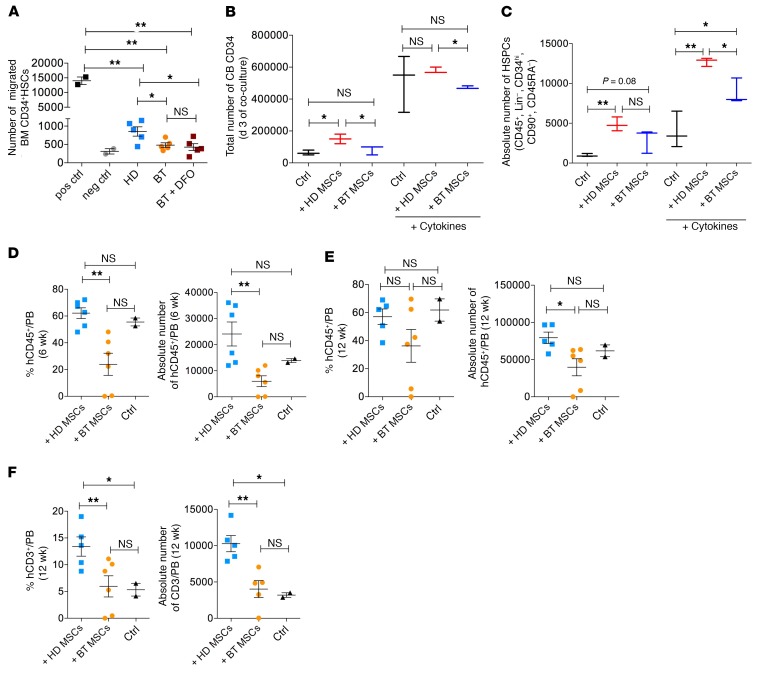
Impaired hematopoietic supportive capacity of BT-MSCs. (**A**) Transwell migration assay of cord blood (CB) CD34^+^ toward HD-MSCs (*n* = 5; blue squares), BT-MSCs (*n* = 5; orange circles), and BT-MSCs (purple squares) treated with 100 μM DFO for 24 hours. Migration capacity is represented as absolute number of CD45^+^ cells migrated into the bottom chamber. Positive control (pos ctrl): SDF-1 (100 ng/ml). Negative control (neg ctrl): basal medium. Each error bar shows mean ± SEM. (**B**) Total number of live cord blood (CB) CD34^+^ cells after 3 days of coculture with HD-MSCs (red) and BT-MSCs (blue) in the presence or absence of proper cytokines. CB CD34^+^ cells cultured for 3 days in the presence or absence of proper cytokines were used as control (black). Data are mean ± SEM (HD: *n* = 3; BT: *n* = 3). (**C**) Absolute number of primitive HSPCs identified as Lin^–^, CD34^hi^, CD90^+^, CD45RA^–^ on CD45^+^ cells after 3 days of coculture with HD-MSCs (red) and BT-MSCs (blue) in the presence or absence of proper cytokines. CB CD34^+^ cells cultured alone in the presence or absence of proper cytokines were used as control (black) (HD: *n* = 3; BT: *n* = 3). (**D**) Percentage (left panel) and absolute number (right panel) of human CD45^+^ cells detected in the peripheral blood of NSG mice 6 weeks after intra tail vein coinfusion of 2.5 × 10^5^ human CB CD34 with 1 × 10^6^ HD- or BT-MSCs. (**E**) Percentage (left panel) and absolute number (right panel) of human CD45^+^ cells detected in the peripheral blood of NSG mice at a later time point (12 weeks). (**F**) Percentage (left panel) and absolute number (right panel) of human T lymphocytes (CD3^+^) detected in the peripheral blood of NSG mice 12 weeks after transplantation. Mice transplanted with CB CD34^+^ cells alone were used as controls. Data are mean ± SEM. In all panels, each dot represents an irradiated mouse transplanted with CB CD34^+^ + HD-MSCs (light blue), CB CD34^+^ + BT-MSCs (orange), or CB CD34^+^ (black). *P* values were determined by Student’s *t* test (*P < 0.05; **P < 0.001).

Most importantly, we asked whether the reduced expression of BM niche–associated genes could be associated with an impairment in the hematopoietic supportive capacities of BT-MSCs in vitro and in vivo. We evaluated the ability of MSCs to promote HSC homing by transwell migration assay. The same amount of BT- and HD-MSCs was seeded in the bottom chamber well. BM-derived HSCs were plated in the upper chamber well in HSC medium conditioned by MSCs for 24 hours. We measured the number of CD34^+^ HSCs that had migrated toward the mesenchymal compartment 3 hours later. We showed that the capacity of BT-MSCs to attract HSCs was reduced in comparison to HD controls (Figure 8A). DFO treatment failed to restore the chemoattractive function of BT-MSCs (Figure 8A). Our preliminary results on histone methylation (Supplemental Figure 4) suggest that prolonged iron exposure in vivo may induce an epigenetic remodeling of the BT-MSC gene expression profile, which could irreversibly affect BT function.

We performed 2D coculture experiments to assess the capacity of BT- and HD-MSCs to sustain expansion and maintenance of primitive HSCs in vitro. After 3 days of coculture, we observed a positive effect of HD-MSCs on cord blood (CB) HSC expansion in the presence of cytokines (hIL6, hTPO, hFlt3, hSCF) (87), whereas BT-MSCs were less efficient in promoting CB CD34^+^ cell amplification (Figure 8B). In the absence of cytokines, CB CD34^+^ cell death was partially prevented by HD-MSCs. This prosurvival effect was less significant when CB CD34^+^ cells were cocultured with BT-MSCs (Figure 8B). Next, we studied the composition of CB CD34^+^ cells by FACS analysis after coculture, with the aim to define the percentage and the absolute number of primitive HSCs defined as CD45^+^, Lin^–^, CD34^hi^, CD90^+^, and CD45RA^–^ on total CD45^+^ cells (Supplemental Figure 6, A and B). We observed a significantly higher enrichment of primitive HSCs in CB CD34^+^ cells cocultured with MSCs, both in the presence and absence of cytokines. HD-MSCs were substantially more efficient than BT-MSCs in promoting the expansion and maintenance of primitive HSCs (Figure 8C). These results confirmed an impaired capacity of BT-MSCs to sustain primitive HSCs in vitro.

We then moved to an in vivo setting to test the ability of BT- and HD-MSCs to facilitate HSC engraftment in a xenogenic transplant model. CB CD34^+^ HSCs were transplanted via intra tail vein injection into sublethally irradiated NOD/SCID/IL-2Rγ null (NSG) mice alone or coinfused with HD- or BT-MSCs. Six weeks after transplantation, peripheral blood samples were analyzed for the presence of hCD45^+^ cells. We detected a significantly lower percentage and absolute number of human donor cells in the peripheral blood of NSG mice receiving the cotransplantation of CB CD34^+^ cells and BT-MSCs, as compared with cotransplantation of CB CD34^+^ cells and HD-MSCs (Figure 8D) (median human hCD45^+^ cells: HDs, 62.15%; BTs, 23.9%; *P* = 0.0019; median absolute number human hCD45^+^ cells: HDs, 24.078 cells; BTs, 5977 cells; *P* = 0.0048). As expected at 6 weeks after HSC transplantation, the vast majority of human CD45^+^ cells were differentiated into B cells (CD13^–^, CD33^–^, CD3^–^, CD19^+^) in both transplantation settings (Supplemental Figure 7A). At a later time point (12 weeks) we observed a similar level of hCD45^+^ cell engraftment in all 3 groups of mice that underwent transplantation (Figure 8E), but a significantly faster immunological reconstitution in terms of recovery of CD3^+^ T cells when HD-MSCs were coinfused with HSCs (median human CD3^+^CD19^–^ cells: HDs, 13.4%; BTs, 5.9%; *P* = 0.023; median absolute number human CD3^+^CD19^–^ cells: HDs, 10.273 cells; BTs, 4026 cells; *P* = 0.0046) (Figure 8F and Supplemental Figure 7B). Altogether, these results indicated that BT-MSCs are less efficient in favoring HSC homing and engraftment in vivo and less capable of accelerating immunological reconstitution after HSC transplantation than HD-MSCs, possibly due to the reduced expression of hematopoietic supportive soluble factors and cytokines. These results are relevant when considering cotransplantation strategies for autologous MSCs and gene-corrected HSCs to facilitate engraftment in BT patients.

Finally, we developed a humanized ossicle model to investigate whether BT-MSCs are capable of forming a proper and supportive BM niche in vivo. As first, gelatin scaffolds preseeded with BT- or HD-MSCs with endothelial cells (HUVECs) were implanted under the skin of nonirradiated NSG mice according to a previously published protocol (88, 89). Five weeks after implantation, humanized ossicles were collected and analyzed by histology. Hematoxylin and eosin staining of the ossicle sections showed the appearance of a bony structure colonized by hematopoietic elements of murine origin in HD-MSC ossicles. Newly formed sinusoidal vessels were also found when HD-MSCs were tested. On the contrary, a delay in the formation of BM structures was observed in BT-MSC–derived ossicles. Few bony cells were observed, with bone lacunae empty and colonized by few hematopoietic cells. A reduced number of vessels was also observed. Immature bone and abundant extracellular matrix were found. These data suggest that BT-MSCs, which are supposed to be fundamental elements in the formation and maintenance of hematopoietic cells in the BM niche, are altered in the ability to form a proper niche in vivo (Supplemental Figure 8, A–C). Next, we implanted gelatin scaffold preseeded with MSCs, HUVECs, and human CD34^+^ HSCs into the flanks of NSG mice to understand whether BT-derived ossicles were capable of sustaining human hematopoiesis. Twelve weeks after implantation, ossicles were collected and analyzed by FACS and immunofluorescence to detect the presence of human HSCs. Importantly, we discovered that the percentage of hCD45^+^ cells detected by FACS in the collagenase-digested ossicles was significantly reduced in BT-derived ossicles compared with HD-derived ossicles (Supplemental Figure 9A). We also analyzed the presence of hCD45^+^ cells by immunofluorescence on HD- and BT-derived ossicle sections (Supplemental Figure 9, C and D) and confirmed a significantly higher number of CD45^+^ cells in HD-derived ossicles as compared with BT-derived ossicles (Supplemental Figure 9B). Human mesenchymal elements were identified by human vimentin expression. These data suggest that BT-MSCs, which are supposed to be fundamental elements in the formation and maintenance of hematopoietic cells in the BM niche, are altered in their ability to form a proper niche in vivo, making them less capable of sustaining HSPC in vivo in the 3D niche model.

## Discussion

In this study, we isolated BM-derived MSCs from patients affected by transfusion-dependent BT and age-matched HDs to investigate phenotypic and functional differences in the BM niche stromal compartment, which could help explain the increased risk of graft rejection and mixed chimerism in these patients after HSC transplantation (90–92). We discovered a significant pauperization of the most primitive MSC pool, which correlated with reduced clonogenic capacity, lower proliferation rate, earlier cell-cycle arrest, and impaired differentiation potential of BT-MSCs compared with HD controls. Our results show that BT-MSCs failed to differentiate into osteoblasts and form bone parallel with the bone disease typical of BT patients, including osteoporosis, growth impairment, spinal deformities, and fragility fractures, which represents an important cause of morbidity (93, 94). Similarly, bone abnormalities have been observed in mouse models of IO (95). In line with our data on ex vivo–expanded MSCs, CD105^+^ MSCs freshly isolated from human BT BM samples showed a limited capacity of osteogenic differentiation (96). Despite several works that described enhanced adipogenesis in MSCs exposed to a high level of ROS (97, 98), we observed a block in the adipogenic differentiation process, possibly due to the reduced presence of multipotent progenitors endowed with the ability to also form adipose tissue. Importantly, the reduced frequency of primitive MSCs is not an in vitro artifact. Indeed, we found a trend in vivo toward a decreased frequency of CD146^+^ cells in BM-MNCs obtained from BT patients as compared with those from HDs. Taking into consideration that CD146^+^ and CD271^+^ MSCs are supposed to reside closely in contact with quiescent HSCs in specific areas of the human BM niche (31, 34–36), any alterations, including their reduced frequency, could potentially introduce questions regarding the composition of the HSC compartment in BT patients and also the capability of the BM stromal compartment in BT patients to sustain HSC functions. In support of this hypothesis, we found a global gene repression of key molecules necessary to sustain hematopoiesis, including *CXCL12*, *ANGPT1*, *VEGFA*, *FGF2*, and *IL6*, that negatively affected the ability of BT-MSCs to attract HSCs in vitro, to sustain their expansion and primitive phenotype in 2D in vitro systems, to favor their engraftment and immunological reconstitution in xenogenic transplant models, and to form a proper BM niche in vivo. These results endorse the evidence of impaired HSC function caused by interaction with an altered BM niche in BT patients (54).

Notably, we demonstrated a situation of iron accumulation in the BM niche of BT patients. Although IO due to chronic blood transfusions has been frequently observed in patients and animal models of BT despite the use of iron chelators (6, 7, 9), here, for what we believe is the first time, we measured total iron and ferritin levels in the plasma of BM aspirates of BT patients before allogeneic HSC transplantation or HSC-GT and showed high levels of ferritin as compared with peripheral blood samples (*P* = 0.05), indicating that the BM niche is a site for accumulation of excessive iron. We demonstrated a direct role of MSCs in managing iron excess. We found that MSCs can uptake iron through iron transporters and *TFR1*, and express ferritin genes for iron storage (Figure 4, B and C). A similar function has been described for macrophages at the systemic level and in the BM (99). However, prolonged iron exposure may alter the iron sensing and storage machinery of MSCs, causing accumulation of cytoplasmic free iron and increasing ROS levels in BT-MSCs (Figure 4, C and D and Figure 5A). In addition, we found an impaired antioxidant response in BT-MSCs, which may get exhausted in repeated and unsuccessful attempts to counteract oxidative stress. In fact, we observed a reduced expression of antioxidant genes in BT-MSCs, an altered activation of ferroportin (*SLC40A1*), and an inappropriate regulation of iron-related genes, such as *TFR1*, likely due to ROS production (100–102). According to our data, reduced antioxidant defenses have been measured in Mediterranean BT patients (103). We demonstrated that increased ROS levels may be responsible for the pauperization of primitive MSCs and for the functional defects of BT-MSCs, as seen in other biological systems (59–61, 104). Although further studies are necessary to dissect the epigenetic state of BT-MSCs, we speculate that iron excess may control the expression of several genes involved in MSC functionality by modulating the activity of iron-dependent chromatin-remodeling proteins (84–86). It is noteworthy that several genes involved in the hematopoietic supportive function of MSCs are specifically subjected to H3K36 regulation, whose methylation is controlled by a family of KDMs robustly expressed in MSCs. A reduced level of methylation correlates with a reduced expression of these genes in BT-MSCs. Importantly, in line with the observations that we made in the small group (*n* = 3) of adult BT patients, we intend to verify in a larger cohort whether the phenotypic and functional characteristics of MSCs isolated from adult patients are more affected compared with pediatric samples, possibly due to more prolonged oxidative stress exposure.

Finally, our results indicate the possibility of targeting the BM niche with the aim of reducing oxidative stress and potentially ameliorating the HSC transplantation outcome. We propose to first correlate BM niche iron levels with the transplant outcome data of BT patients undergoing both allogeneic HSCT or HSC-GT with the aim to look for possible associations between BM IO state and risk of graft failure and development of mixed chimerism (90, 91). If this is confirmed, pretransplantation iron and ferritin levels in the BM plasma of BT patients may be employed in the future as potential biomarkers to estimate the degree of IO. Because of oxidative stress in the niche, it must be considered that the higher the level of iron accumulation, the higher the risk might be to transplant HSCs into an altered BM niche (92). We believe that future interventions on the BM niche of BT patients are needed to diminish IO and oxidative stress, potentially affecting HSC transplantation outcome. In particular, our study may support the development of new biological conditioning strategies in BT patients with the aim to avoid further damage to the BM niche caused by chemotherapeutic agents (105), and suggests that the use of conventional iron chelators is not sufficient to control iron accumulation in the BT BM niche, possibly because the iron chelators cannot properly reach this environment. Local delivery into the niche of iron chelators and/or antioxidants before the transplantation may represent a strategy to reestablish a less inflamed and more functional microenvironment and therefore eventually ameliorate transplantation outcome. Systemic administration of antiinflammatory and/or antioxidant drugs before the transplant procedure might also be considered to reach the same objective, being more clinically feasible. In conclusion, we found an impairment in the biological and functional properties of the mesenchymal compartment of BT patients that affected MSC ability to properly sustain HSC functions in vitro and in vivo. We believe that a profound knowledge of the biology of the BM microenvironment is fundamental for the optimization of future transplantation strategies, especially in the context of HSC-GT, where the relationship between HSCs and the stroma may influence harvest and behavior in culture, as well as their engraftment kinetics after gene modification (106).

## Methods

### Isolation and culture of BM-derived MSCs.

Eleven BT patients were included in the study: 3 adults and 8 children. All patients were on a regular blood transfusion regimen and iron-chelation therapy with deferasirox. They all had a high pretransplantation transfusion requirement; comorbidities were mild. Patient BM was collected before HSC-GT or allogeneic HSCT, after obtaining patient or parental informed consent according to a protocol approved by the San Raffaele Ethical Committee. Control MSCs were obtained from residual BM cells of 9 age-matched HDs who donated BM for transplantation at San Raffaele Scientific Institute. MSCs were isolated according to a previously published protocol (55). MSCs were cultured in DMEM+GlutaMAX (Thermo Fisher Scientific, catalog 10566-016) supplemented with 5% platelet lysate and 1% penicillin/streptomycin.

### Fibroblast colony-forming unit assay.

CD34^–^ MNCs were plated at a density of 1 × 10^5^ cells/cm^2^ in basal medium. Colony-forming units were stained with 1% crystal violet (Sigma-Aldrich, catalog C0775) and manually counted after 7 days.

### Population doubling time.

MSCs were plated at a density of 3 × 10^4^ cells/cm^2^, detached, and counted at a confluence of 80%–90% using Trypan blue to distinguish live cells. Proliferative capacity was calculated as population doubling time according to http://www.doubling-time.com/compute.php Cell proliferation was followed from passage 3 to 7.

### In vitro adipogenic and osteogenic differentiation of MSCs.

For adipogenic and osteogenic differentiation, MSCs were cultured in proper induction medium according to a previously published protocol (55). Differentiation was evaluated after 21 days by proper staining and reverse transcriptase quantitative PCR (RT-qPCR). Adipogenic differentiation medium was made up of alpha MEM (Thermo Fisher Scientific), 1% penicillin/streptomycin, and 10% MesenCult MSC Stimulatory Supplement (STEMCELL), supplemented with 10^−10^ M dexamethasone, 50 μg/ml l-ascorbic acid, 10 μg/ml insulin, 5 μM 3-isobutyl-1-methylxanthine, 0.2^−10^ M indomethacin, and 0.5 mM β-glycerol phosphate (Sigma-Aldrich). Osteogenic differentiation medium was made up of alpha MEM, 1% penicillin/streptomycin, and 10% MesenCult MSC Stimulatory Supplement, supplemented with 10^−10^ M dexamethasone and 50 μg/ml l-ascorbic acid. Starting from day 7 of differentiation, 5 mM β-glycerol phosphate was added to the medium.

### ROS measurement.

ROS levels were measured using the Cell Rox Reagent (Thermo Fisher Scientific, catalog C10444) in HD- and BT-MSCs at the basal level or after 5 days of iron treatment, according to the manufacturer’s instructions. Cell viability was analyzed by using 7AAD (BD Biosciences, catalog 559925) prior to analysis.

### Iron tests.

Determination of total iron (μg/dl) was performed on plasma samples from HD and BT BM aspirates using ferrozine-based reagent without deproteinization on COBAS C 8000 WKC/MET/027 (Roche). Determination of ferritin (ng/ml) was performed on plasma samples from HD and BT BM aspirates by electrochemiluminescence immunoassay (ECLIA) on COBAS C 8000 WKC/MET/027.

### Perl’s staining.

Ferric iron was detected in HD and BT samples plated into chamber slide (Lab-Tek, catalog 154461) using the iron stain kit (Abcam, catalog ab150674), according to the manufacturer’s instructions. Slides were mounted using the FluorSave Reagent for microscopic evaluation (Nikon, Eclipse Ni-U + DS-Fi-2).

### Transwell migration assay.

HD- and BT-MSCs (1.5 × 10^4^) were plated into a 24-well plate for transwell migration assay. BM-HSCs (Lonza, catalog 2M-101C) were thawed in Stem Cell growth medium (CellGenix) supplemented with 1% penicillin/streptomycin, 1% glutamine, and proper cytokines (100 ng/ml hTPO, 300 ng/ml hFLT3, 300 ng/ml hSCF, 60 ng/ml hIL3) for expansion. The MSC basal medium was replenished 24 hours before starting the migration assay using HSC proper medium without additional cytokines for conditioning. BM CD34^+^ HSCs were collected, counted, and plated (1.5 × 10^5^) into the transwell insert (Corning, catalog CLS3421) in 200 μl stem cell growth medium without any additional cytokines. Three hours later, cells in the bottom compartment were collected and stained with human CD45^+^ antibody (BD Biosciences, catalog 340910). The number of BM HSCs that migrated toward the bottom compartment was calculated as percentage of CD45^+^ cells on total number of seeded HSCs using the BD Accuri C6 flow cytometer, which allows direct calculation of cell concentration per microliter for each population of interest in a certain fixed time frame (2 minutes). Experiments were performed in duplicate for each sample (5 HD-MSCs and 5 BT-MSCs). Stem Cell growth medium containing 100 ng SDF-1 (Peprotech) was used as positive control. Cells were exposed to 100 μM DFO for 24 hours before performing the assay. Stem Cell growth medium without any additional cytokines or cells was used as negative control.

### 2D coculture of CB HSCs and MSCs.

HD- and BT-MSCs were plated at a density of 7000 cells/cm^2^ and expanded for 3 days. Twenty-four hours before starting the coculture experiment, CB CD34^+^ HSCs (Lonza, catalog 2C-101) were thawed in Stem Cell growth medium (CellGenix, catalog 20802-0500) supplemented with 1% penicillin/streptomycin, 1% glutamine, and proper cytokines (20 ng/ml hTPO, 100 ng/ml hFLT3, 100 ng/ml hSCF, 20 ng/ml hIL6) for expansion. MSC basal medium was replenished with HSC medium with or without proper cytokines for conditioning. CB CD34^+^ cells (2 × 10^5^) were plated in 200 μl MSC conditioned medium on a MSC layer. After 3 days of coculture, CB CD34^+^ cells were collected by pipetting and live cells were counted using Trypan blue. The percentage of primitive CB CD34^+^ cells was evaluated by flow cytometry analysis. Cells were incubated with the following antibody mix: CD16 PE (BD Biosciences, catalog 332779), CD14 PE (BioLegend, catalog 301806) CD3 PE (BD Biosciences, catalog 345765), CD15 PE (BioLegend, catalog 301906), CD56 PE (BD Biosciences, catalog 345812), CD19 PE (BD Biosciences, catalog 345789), CD34 BV421 (BioLegend, catalog 343610), CD45RA APC-H7 (BioLegend, catalog 304128), CD45 BUV395 (BD Biosciences, catalog 563792), and CD90 APC (BD Biosciences, catalog 559869) for 10 minutes at room temperature in the dark. After washing with PBS plus 2% FBS, cells were centrifuged for 5 minutes at 1500 rpm and resuspended into 100 μl PBS plus 2% FBS. The capacity of MSCs to maintain primitive HSCs in vitro was evaluated by flow cytometry analysis using the BD LSRFortessa. Primitive CB CD34^+^ cells were defined as CD45^+^, Lin^–^, CD34^hi^, CD45RA^–^, CD90^+^. Unstained cells were used as a negative control. Flow cytometry assay was standardized using SPHERO Rainbow Calibration Particles (8 peaks) (Spherotech). Analysis of flow cytometry results was performed using FlowJo software (Tree Star). Experiments were performed in triplicate for each sample (3 HD MSCs and 3 BT MSCs). CB CD34^+^ cells alone cultured in Stem Cell growth medium with or without cytokines were used as controls.

### Transplantation of human CB CD34^+^ cells and MSCs into NSG mice.

NSG mice were purchased from Charles River Laboratories and maintained in a specific pathogen–free animal facility. Adult female mice (7 weeks old) were sublethally irradiated (200 Gy) and transplanted via intra tail vein injection with 2.5 × 10^5^ human CB CD34^+^ cells (mix from 4 different donors) alone or in combination with 1 × 10^6^ human MSCs (mix from 6 different HDs or 6 different BT patients). Peripheral blood samples were collected 6 and 12 weeks after human cell coinfusion. All procedures were performed according to protocols approved by the Committee for Animal Care and Use of San Raffaele Scientific Institute. Whole blood counts were determined on 10 μl total blood using a Sysmex KX-21N hemocytometer. The presence of human cells was determined by flow cytometry using the following antibody mix: hCD45 APC (BD Biosciences, catalog 304012), hCD33 Vio-Blue (Miltenyi Biotec, catalog 130-099-485), hCD13 PerCP Cy5.5 (BD Biosciences, catalog 561361), hCD19 PE Cy7 (BioLegend, catalog 302216), and hCD3 PE (BD Biosciences, catalog 345765). Red blood cells were lysed using the Beckman Coulter TQ-PREP workstation. All samples were run on a BD Biosciences FACSCanto II cytometer. At least 10,000 were recorded. Flow cytometry results were analyzed using FlowJo software.

### Statistics.

All data are mean ± SEM. Statistical analysis was carried out using the Prism software (GraphPad). For statistical comparison, an unpaired, 2-tailed Student’s *t* test was used. All the data meet the assumption of the statistical tests used. A *P* value less than 0.05 was considered significant.

### Study approval.

Human BM samples were collected after obtaining patient or parental informed consent according to the research protocol TIGET09 approved by the Ethical Committee of the San Raffaele Hospital, Milan. Animal studies were approved by the San Raffaele Scientific Institute and IACUC (number 648) and performed according to the Italian Ministry of Health guidelines for the use and the care of experimental animals.

## Author contributions

SC designed the study, performed most of the experiments, analyzed results and wrote the paper. VR, SR, and SP performed experiments and collected the data. A Aprile and LS contributed to the analyses of the results. SS purified CD34^–^ cell fraction from bone marrow aspirates and helped with critical discussion. MAA performed the immunomodulation experiments. RJH and LBR performed some of the in vivo experiments and flow cytometry analysis. MZ and SM critically read the manuscript. A Aiuti, GF, and FC critically read the manuscript and helped with scientific discussion. MEB advised on the study design, analyzed data, and contributed to the final writing of the paper.

## Supplementary Material

Supplemental data

## Version 1 02/25/2019

Electronic publication

## Version 2 04/01/2019

Print issue publication

## References

[1] Williams TN, Weatherall DJ (2012). World distribution, population genetics, and health burden of the hemoglobinopathies. Cold Spring Harb Perspect Med.

[2] Weatherall DJ (2001). Phenotype-genotype relationships in monogenic disease: lessons from the thalassaemias. Nat Rev Genet.

[3] Thein SL (2013). The molecular basis of β-thalassemia. Cold Spring Harb Perspect Med.

[4] Taher AT, Weatherall DJ, Cappellini MD (2018). Thalassaemia. Lancet.

[5] Higgs DR, Engel JD, Stamatoyannopoulos G (2012). Thalassaemia. Lancet.

[6] Saliba AN, Harb AR, Taher AT (2015). Iron chelation therapy in transfusion-dependent thalassemia patients: current strategies and future directions. J Blood Med.

[7] Bou-Fakhredin R, Bazarbachi AH, Chaya B, Sleiman J, Cappellini MD, Taher AT (2017). Iron overload and chelation therapy in non-transfusion dependent thalassemia. Int J Mol Sci.

[8] Borgna-Pignatti C (2005). Survival and complications in thalassemia. Ann N Y Acad Sci.

[9] Taher AT, Saliba AN (2017). Iron overload in thalassemia: different organs at different rates. Hematology Am Soc Hematol Educ Program.

[10] Vento S, Cainelli F, Cesario F (2006). Infections and thalassaemia. Lancet Infect Dis.

[11] Zhang Y (2015). Effects of iron overload on the bone marrow microenvironment in mice. PLoS One.

[12] Lekawanvijit S, Chattipakorn N (2009). Iron overload thalassemic cardiomyopathy: iron status assessment and mechanisms of mechanical and electrical disturbance due to iron toxicity. Can J Cardiol.

[13] Angelucci E (2010). Hematopoietic stem cell transplantation in thalassemia. Hematology Am Soc Hematol Educ Program.

[14] Angelucci E (2014). Hematopoietic stem cell transplantation in thalassemia major and sickle cell disease: indications and management recommendations from an international expert panel. Haematologica.

[15] Bernardo ME (2012). Allogeneic hematopoietic stem cell transplantation in thalassemia major: results of a reduced-toxicity conditioning regimen based on the use of treosulfan. Blood.

[16] Baronciani D (2016). Hemopoietic stem cell transplantation in thalassemia: a report from the European Society for Blood and Bone Marrow Transplantation Hemoglobinopathy Registry, 2000-2010. Bone Marrow Transplant.

[17] La Nasa G (2005). Unrelated bone marrow transplantation for beta-thalassemia patients: The experience of the Italian Bone Marrow Transplant Group. Ann N Y Acad Sci.

[18] Lucarelli G, Gaziev J (2008). Advances in the allogeneic transplantation for thalassemia. Blood Rev.

[19] Rachmilewitz EA, Giardina PJ (2011). How I treat thalassemia. Blood.

[20] Arumugam P, Malik P (2010). Genetic therapy for beta-thalassemia: from the bench to the bedside. Hematology Am Soc Hematol Educ Program.

[21] Breda L (2010). A preclinical approach for gene therapy of beta-thalassemia. Ann N Y Acad Sci.

[22] Negre O (2015). Preclinical evaluation of efficacy and safety of an improved lentiviral vector for the treatment of β-thalassemia and sickle cell disease. Curr Gene Ther.

[23] Miccio A (2008). In vivo selection of genetically modified erythroblastic progenitors leads to long-term correction of beta-thalassemia. Proc Natl Acad Sci U S A.

[24] Roselli EA (2010). Correction of beta-thalassemia major by gene transfer in haematopoietic progenitors of pediatric patients. EMBO Mol Med.

[25] Ferrari G, Cavazzana M, Mavilio F (2017). Gene therapy approaches to hemoglobinopathies. Hematol Oncol Clin North Am.

[26] Frisch BJ (2019). The hematopoietic stem cell niche: what’s so special about bone?. Bone.

[27] Crane GM, Jeffery E, Morrison SJ (2017). Adult haematopoietic stem cell niches. Nat Rev Immunol.

[28] Morrison SJ, Scadden DT (2014). The bone marrow niche for haematopoietic stem cells. Nature.

[29] Omatsu Y (2010). The essential functions of adipo-osteogenic progenitors as the hematopoietic stem and progenitor cell niche. Immunity.

[30] Méndez-Ferrer S (2010). Mesenchymal and haematopoietic stem cells form a unique bone marrow niche. Nature.

[31] Asada N, Takeishi S, Frenette PS (2017). Complexity of bone marrow hematopoietic stem cell niche. Int J Hematol.

[32] Asada N (2017). Differential cytokine contributions of perivascular haematopoietic stem cell niches. Nat Cell Biol.

[33] Battula VL (2009). Isolation of functionally distinct mesenchymal stem cell subsets using antibodies against CD56, CD271, and mesenchymal stem cell antigen-1. Haematologica.

[34] Quirici N, Soligo D, Bossolasco P, Servida F, Lumini C, Deliliers GL (2002). Isolation of bone marrow mesenchymal stem cells by anti-nerve growth factor receptor antibodies. Exp Hematol.

[35] Tormin A (2011). CD146 expression on primary nonhematopoietic bone marrow stem cells is correlated with in situ localization. Blood.

[36] Sacchetti B (2007). Self-renewing osteoprogenitors in bone marrow sinusoids can organize a hematopoietic microenvironment. Cell.

[37] Li H, Ghazanfari R, Zacharaki D, Lim HC, Scheding S (2016). Isolation and characterization of primary bone marrow mesenchymal stromal cells. Ann N Y Acad Sci.

[38] Friedenstein AJ, Chailakhjan RK, Lalykina KS (1970). The development of fibroblast colonies in monolayer cultures of guinea-pig bone marrow and spleen cells. Cell Tissue Kinet.

[39] Caplan AI (1991). Mesenchymal stem cells. J Orthop Res.

[40] Bianco P (2007). Self-renewing mesenchymal progenitors in the bone marrow and in other mesodermal tissues. J Stem Cells Regen Med.

[41] Beyer Nardi N, da Silva Meirelles L (2006). Mesenchymal stem cells: isolation, in vitro expansion and characterization. Handb Exp Pharmacol.

[42] Dominici M (2006). Minimal criteria for defining multipotent mesenchymal stromal cells. The International Society for Cellular Therapy position statement. Cytotherapy.

[43] Bartholomew A (2002). Mesenchymal stem cells suppress lymphocyte proliferation in vitro and prolong skin graft survival in vivo. Exp Hematol.

[44] Bernardo ME, Fibbe WE (2013). Mesenchymal stromal cells: sensors and switchers of inflammation. Cell Stem Cell.

[45] Nauta AJ, Fibbe WE (2007). Immunomodulatory properties of mesenchymal stromal cells. Blood.

[46] Bernardo ME (2011). Co-infusion of ex vivo-expanded, parental MSCs prevents life-threatening acute GVHD, but does not reduce the risk of graft failure in pediatric patients undergoing allogeneic umbilical cord blood transplantation. Bone Marrow Transplant.

[47] Le Blanc K (2008). Mesenchymal stem cells for treatment of steroid-resistant, severe, acute graft-versus-host disease: a phase II study. Lancet.

[48] de Lima M (2012). Cord-blood engraftment with ex vivo mesenchymal-cell coculture. N Engl J Med.

[49] Devine SM, Hoffman R (2000). Role of mesenchymal stem cells in hematopoietic stem cell transplantation. Curr Opin Hematol.

[50] Koç ON (2000). Rapid hematopoietic recovery after coinfusion of autologous-blood stem cells and culture-expanded marrow mesenchymal stem cells in advanced breast cancer patients receiving high-dose chemotherapy. J Clin Oncol.

[51] Lazarus HM (2005). Cotransplantation of HLA-identical sibling culture-expanded mesenchymal stem cells and hematopoietic stem cells in hematologic malignancy patients. Biol Blood Marrow Transplant.

[52] Chai X (2015). ROS-mediated iron overload injures the hematopoiesis of bone marrow by damaging hematopoietic stem/progenitor cells in mice. Sci Rep.

[53] Okabe H, Suzuki T, Uehara E, Ueda M, Nagai T, Ozawa K (2014). The bone marrow hematopoietic microenvironment is impaired in iron-overloaded mice. Eur J Haematol.

[54] Aprile A, Lidonnici MR, Gulino A, Tripodo C, Mandelli G, Ferrari G (2015). Alteration of HSC functions in thalassemia. Blood.

[55] Ingo DM (2016). Bone marrow-derived CD34- fraction: a rich source of mesenchymal stromal cells for clinical application. Cytotherapy.

[56] Colter DC, Sekiya I, Prockop DJ (2001). Identification of a subpopulation of rapidly self-renewing and multipotential adult stem cells in colonies of human marrow stromal cells. Proc Natl Acad Sci U S A.

[57] Mabuchi Y (2013). LNGFR(+)THY-1(+)VCAM-1(hi+) cells reveal functionally distinct subpopulations in mesenchymal stem cells. Stem Cell Reports.

[58] Pal B, Das B (2017). In vitro culture of naïve human bone marrow mesenchymal stem cells: a stemness based approach. Front Cell Dev Biol.

[59] Bigarella CL, Liang R, Ghaffari S (2014). Stem cells and the impact of ROS signaling. Development.

[60] Chaudhari P, Ye Z, Jang YY (2014). Roles of reactive oxygen species in the fate of stem cells. Antioxid Redox Signal.

[61] Shao L, Li H, Pazhanisamy SK, Meng A, Wang Y, Zhou D (2011). Reactive oxygen species and hematopoietic stem cell senescence. Int J Hematol.

[62] Urao N, Ushio-Fukai M (2013). Redox regulation of stem/progenitor cells and bone marrow niche. Free Radic Biol Med.

[63] MacKenzie EL, Iwasaki K, Tsuji Y (2008). Intracellular iron transport and storage: from molecular mechanisms to health implications. Antioxid Redox Signal.

[64] Nemeth E (2004). Hepcidin regulates cellular iron efflux by binding to ferroportin and inducing its internalization. Science.

[65] Fang S (2018). Effects of intracellular iron overload on cell death and identification of potent cell death inhibitors. Biochem Biophys Res Commun.

[66] Barisani D, Meneveri R, Ginelli E, Cassani C, Conte D (2000). Iron overload and gene expression in HepG2 cells: analysis by differential display. FEBS Lett.

[67] Brissot P, Ropert M, Le Lan C, Loréal O (2012). Non-transferrin bound iron: a key role in iron overload and iron toxicity. Biochim Biophys Acta.

[68] Anderson CP, Shen M, Eisenstein RS, Leibold EA (2012). Mammalian iron metabolism and its control by iron regulatory proteins. Biochim Biophys Acta.

[69] Beattie L (2016). Bone marrow-derived and resident liver macrophages display unique transcriptomic signatures but similar biological functions. J Hepatol.

[70] Yuan XM, Li W, Baird SK, Carlsson M, Melefors O (2004). Secretion of ferritin by iron-laden macrophages and influence of lipoproteins. Free Radic Res.

[71] Ludin A (2014). Reactive oxygen species regulate hematopoietic stem cell self-renewal, migration and development, as well as their bone marrow microenvironment. Antioxid Redox Signal.

[72] Pessina A (2015). Drug-releasing mesenchymal cells strongly suppress B16 lung metastasis in a syngeneic murine model. J Exp Clin Cancer Res.

[73] Conforti A (2014). Human mesenchymal stromal cells primed with paclitaxel, apart from displaying anti-tumor activity, maintain their immune regulatory functions in vitro. Cytotherapy.

[74] Pascucci L (2014). Paclitaxel is incorporated by mesenchymal stromal cells and released in exosomes that inhibit in vitro tumor growth: a new approach for drug delivery. J Control Release.

[75] Donovan A (2005). The iron exporter ferroportin/Slc40a1 is essential for iron homeostasis. Cell Metab.

[76] Ward DM, Kaplan J (2012). Ferroportin-mediated iron transport: expression and regulation. Biochim Biophys Acta.

[77] Zhang Y (2016). CXCR4/CXCL12 axis counteracts hematopoietic stem cell exhaustion through selective protection against oxidative stress. Sci Rep.

[78] Sugiyama T, Kohara H, Noda M, Nagasawa T (2006). Maintenance of the hematopoietic stem cell pool by CXCL12-CXCR4 chemokine signaling in bone marrow stromal cell niches. Immunity.

[79] Arai F, Hosokawa K, Toyama H, Matsumoto Y, Suda T (2012). Role of N-cadherin in the regulation of hematopoietic stem cells in the bone marrow niche. Ann N Y Acad Sci.

[80] Mendelson A, Frenette PS (2014). Hematopoietic stem cell niche maintenance during homeostasis and regeneration. Nat Med.

[81] Tamma R, Ribatti D (2017). Bone niches, hematopoietic stem cells, and vessel formation. Int J Mol Sci.

[82] Pricola KL, Kuhn NZ, Haleem-Smith H, Song Y, Tuan RS (2009). Interleukin-6 maintains bone marrow-derived mesenchymal stem cell stemness by an ERK1/2-dependent mechanism. J Cell Biochem.

[83] Coutu DL, Galipeau J (2011). Roles of FGF signaling in stem cell self-renewal, senescence and aging. Aging (Albany NY).

[84] Pogribny IP (2013). Modulation of intracellular iron metabolism by iron chelation affects chromatin remodeling proteins and corresponding epigenetic modifications in breast cancer cells and increases their sensitivity to chemotherapeutic agents. Int J Oncol.

[85] Klose RJ, Kallin EM, Zhang Y (2006). JmjC-domain-containing proteins and histone demethylation. Nat Rev Genet.

[86] Teperino R, Schoonjans K, Auwerx J (2010). Histone methyl transferases and demethylases; can they link metabolism and transcription?. Cell Metab.

[87] Genovese P (2014). Targeted genome editing in human repopulating haematopoietic stem cells. Nature.

[88] Passaro D, Abarrategi A, Foster K, Ariza-McNaughton L, Bonnet D (2017). Bioengineering of humanized bone marrow microenvironments in mouse and their visualization by live imaging. J Vis Exp.

[89] Abarrategi A (2017). Versatile humanized niche model enables study of normal and malignant human hematopoiesis. J Clin Invest.

[90] Fouzia NA (2018). Long-term outcome of mixed chimerism after stem cell transplantation for thalassemia major conditioned with busulfan and cyclophosphamide. Bone Marrow Transplant.

[91] Gaziev J (2016). Optimal outcomes in young class 3 patients with thalassemia undergoing HLA-identical sibling bone marrow transplantation. Transplantation.

[92] Yang T, Wu X, Xiao M, Zhang Y, Chen Z, Hu J (2017). Iron Overload results in poor graft function after allogeneic hematopoietic stem cell transplantation by impairing hematopoiesis. Blood.

[93] Piga A (2017). Impact of bone disease and pain in thalassemia. Hematology Am Soc Hematol Educ Program.

[94] De Sanctis V (2018). Bone disease in β thalassemia patients: past, present and future perspectives. Metab Clin Exp.

[95] Tsay J (2010). Bone loss caused by iron overload in a murine model: importance of oxidative stress. Blood.

[96] Hongeng S (2011). Use of in vivo gene expression of isolated bone marrow mesenchymal stromal cells to study the pathophysiology of osteoporosis in patients with severe thalassemia. J Pediatr Hematol Oncol.

[97] Atashi F, Modarressi A, Pepper MS (2015). The role of reactive oxygen species in mesenchymal stem cell adipogenic and osteogenic differentiation: a review. Stem Cells Dev.

[98] Kanda Y, Hinata T, Kang SW, Watanabe Y (2011). Reactive oxygen species mediate adipocyte differentiation in mesenchymal stem cells. Life Sci.

[99] Soares MP, Hamza I (2016). Macrophages and iron metabolism. Immunity.

[100] Gammella E, Recalcati S, Cairo G (2016). Dual role of ROS as signal and stress agents: iron tips the balance in favor of toxic effects. Oxid Med Cell Longev.

[101] Cairo G, Recalcati S (2007). Iron-regulatory proteins: molecular biology and pathophysiological implications. Expert Rev Mol Med.

[102] Yamanaka K (2003). Identification of the ubiquitin-protein ligase that recognizes oxidized IRP2. Nat Cell Biol.

[103] Tsamesidis I (2017). Total antioxidant capacity in Mediterranean β-thalassemic patients. Adv Clin Exp Med.

[104] Hu Q (2018). Oxidative stress promotes exit from the stem cell state and spontaneous neuronal differentiation. Oncotarget.

[105] Aiuti A, Naldini L (2016). Safer conditioning for blood stem cell transplants. Nat Biotechnol.

[106] Cavazzana M, Ribeil JA, Lagresle-Peyrou C, André-Schmutz I (2017). Gene therapy with hematopoietic stem cells: the diseased bone marrow’s point of view. Stem Cells Dev.

