# Supplementary material for: Bone marrow stromal cells from β-thalassemia patients have impaired hematopoietic supportive capacity
Source: J Clin Invest. 2019 Feb 25;129(4):1566–80. doi: 10.1172/JCI123191 (PMC6436882; doi:10.1172/JCI123191)
Supplement: Supplemental data [file jci-129-123191-s019.pdf]

Supplementary Materials:

Materials and Methods

RNA extraction, RT-PCR and real-time PCR

Total RNA was extracted using RNeasy Micro Kit (Qiagen, 74004) according to the manufacturer's instructions. DNase treatment was performed on RNA samples using RNase-Free DNase kit (Qiagen,79254). cDNA was synthesized from 1 µg total RNA using the high capacity reverse transcription kit (Applied Biosystems, 4319983). SYBR Green based quantitative PCR was performed using QuantiFast SYBR Green PCR Kit (Qiagen, 1039712) starting from 5 ng of cDNA with a Viia7 real-time PCR system (Thermofisher). The following primers were used: *ACTNB* F: ACAGAGCCTCGCCTTTGC, *ACTNB* R: ACTCCATGCCCAGGAAGGAA; *PPARg* F: TCAGAAATGCCTTGCAGTGG, *PPARg* R: TATCACTGGAGATCTCCGCCAA; *LPL* F: CCGCCGACCAAAGAAGAGAT, *LPL* R: TAGCCACGGACTCTGCTACT; *FABP4* F: AAACTGGTGGTGGAATGCGT, *FABP4* R: GCGAACTTCAGTCCAGGTCA; *RUNX2* F: CCGGAATGCCTCTGCTGTTA, *RUNX2* R: TGTCTGTGCCTTCTGGGTTC; *SPARC* F: ATTGACGGGTACCTCTCCCA, *SPARC* R: GAAAAAGCGGGTGGTGCAAT; *COL1A2* F: ACAAGGCATTCGTGGCGATA, *COL1A2* R: ACCATGGTGACCAGCGATAC; *SPARC* F: ATTGACGGGTACCTCTCCCA, *SPARC* R: GAAAAAGCGGGTGGTGCAAT; *CD146* F: CAGGGAAGCAGGAGATCACG, *CD146* R: CAGGAGGCCCATCTCTTCTG; *CD271* F: GTATTCCGACGAGGCCAACC, *CD271* R: CCAGGGATCTCCTCGCACT; *DMT1* F: TCTTCTGTTCAGGACCCAGC, *DMT1* R: GGCGTGTCAGGTGGTTG; *ZIP14* F: GCAGCTTCATGGTGACTGAA, *ZIP14* R: GCTAAGCTGCTTCTGCCG; *ZIP8* F: GGCCCCTTCAAACAGGTACA, *ZIP8* R: TGCTGTCACAGAAGCTAATGG; *TFR1* F: TGCTGTCCAGCAGCCATAGG, *TFR1* R: CACCAAACAAGTTAGAGAATGCTGA; *FTL* F: GGACCCCCATCTCTGTGACT; *FTL* R: AGTCGTGCTTGAGAGTGAGC; *FTH* F: AGGTGCGCCAGAACTACCA, *FTH* R: TCGCGGTCAAAGTAGTAAGACATGG; *SOD1* F:

ACTGGTGGTCCATGAAAAAGC, *SOD1* R: AACGACTTCCAGCGTTTCCT; *GSS* F: AAAGGCGAACTAGTGTTGGGA, *GSS* R: AGAGCGTGAATGGGGCATAG; *HMOX1* F: ACTGCGTTCCTGCTCAACAT, *HMOX1* R: GGGGCAGAATCTTGCACTTT; *SLC40A1* F: GATCCTTGGCCGACTACCTG, *SLC40A1* R: CACATCCGATCTCCCCAAGT; *SCF* F: CCCAGAACCCAGGCTCTTTA, *SCF* R: TTTGGCCTTCCCTTTCTCAGG; *CXCL12* F: TGCCCTTCAGATTGTAGCCC, *CXCL12* R: CGAGTGGGTCTAGCGGAAAG; *N-CAD* F: TCACACTGGCAAACCTTCAC, *N-CAD* R: GAAATTGGACCATCACTCGG; *VCAM-1* F: GACCTTCATCCCTACCATTG, *VCAM-1* R: TTCACAGAACTGCCTTCCTCCA; *ANGPT1* F: ACATGGGCAATGTGCCTACA, *ANGPT1* R: TCTCAAGTTTTTGCAGCCACTG; *VEGFA* F: CATCTTCAAGCCATCCTGTG, *VEGFA* R: GGAAGCTCATCTCTCCTATG; *FGF2* F: GCTGTACTGCAAAAACGGGG, *FGF2* R: TAGCTTGATGTGAGGGTCGC; *IL6* F: GATGGATGCTTCCAATCTGG, *IL6* R: TGTTCTGGAGGTACTCTAGG; *KDM2A* F: CGAAGCCTCACACTATGAAACC, *KDM2A* R: TTCTGATCCACCTCTCCACAGA; *KDM2B* F: GACCGGGAAACAAAAGCGTG, *KDM2B* R: TGTCCCGGTTCATCTTCTGC; *KDM3A* F: TGCCAACTCTCCACCTAACCTT, *KDM3A* R: GTCTTAGGCTGAGTACAGCT; *KDM5A* F: CTACGCGGCGGAGTTCG, *KDM5A* R: GAGGCTGCCAGTCCTTGG; *KDM5B* F: AAGATGGGGTTTGCTCCTGG, *KDM5B* R: GCTTCTGCAAACACCTTAGGC. Raw data (Ct values) were analyzed according to the comparative Ct method. Briefly, technical triplicates of three biological replicates were used to validate and determine sample Ct mean. Ct means were processed as follows: ΔCt = (Ct gene of interest - Ct internal control). Level of gene expression was calculated as relative to Actnb = 2−ΔCT sample or as expression fold change = 2−ΔCT sample A / 2−ΔCT sample B; whereby sample B serves for normalizing all samples to be compared.

Adipogenic and osteogenic differentiation staining

For Oil Red O staining, cells were fixed with 4% PFA, washed with water and isopropanol and stained with Oil Red O solution for 15 min. After washing, cells were counterstained with

hematoxylin and fixed with FlourSave Reagent (Merck, 345789) for microscopic evaluation (ZEISS Axio Imager 2) of adipogenic differentiation.

Osteogenic differentiation was evaluated after 21 days by Alizarin Red S staining (Sigma-Aldrich, A5533) and RT-qPCR. For Alizarin Red S staining, cells were fixed with 4% PFA, washed with water, stained with Alizarin Red solution and fixed with FlourSave Reagent for microscopic evaluation of osteogenic differentiation.

Wester Blotting

Except for nuclear-cytoplasmic fractionation, Western blots were performed on protein extract from cells lysed in RIPA buffer (20 mM Tris pH 8, 50 mM NaCl, 0.5% Nadeoxycholate, 0.1% SDS, 1 mM Na3VO4, protease inhibitor cocktail (Roche, 04693116001)) at 4°C for 20 minutes. Following benzonase (Sigma-Aldrich, E1014) treatment, samples were centrifuged for 15 min 14000 rpm at 4°C. Supernatant protein concentrations were determined by Pierce BCA Protein Assay Kit (ThermoFisher, 23225). Lysates were resolved on SDS-PAGE under reducing conditions, and proteins were transferred to a PVDF membrane (ThermoFisher, 88518). For nuclear and cytoplasmic fractionation, cells were lysed in EMSA Buffer A (20 mM Hepes pH 7.6, 20% glycerol, 10 mM NaCl, 1.5 mM MgCl2, 0.2 mM EDTA, 1 mM DTT, 0.1% NP-40) 10 min at 4°C. The cytoplasmic fraction was collected after centrifugation for 5 min at 2500 rpm at 4°C. Following 3 times washing with EMSA Buffer A, nuclei were lysed in EMSA Buffer B (20 mM Hepes pH 7.6, 20% glycerol, 500 mM NaCl, 1.5 mM MgCl2, 0.2mM EDTA, 1 mM DTT, 0.1% NP-40) 30 min at 4°C. Nuclear extracts were collected after centrifugation for 15 min at 13000 rpm at 4°C. The following antibodies were used: anti PCNA (Abcam, ab29, 1:1000), anti total histone H3 (Abcam, ab1791, 1:1000), anti H3K9me3 (Abcam, ab8898, 1:1000), anti H3K36me3 (Abcam, ab9050, 1:300), anti-ferritin (Sigma-Aldrich, F5012), anti p53 (Abcam, ab26, 1:1000) and anti-β-tubulin (Santa Cruz Biotechnology, H-235, 1:2000). Western Lightning Plus ECL HRP substrate

(Perkin Elmer, NEL103001EA) was used, according to manufacturer's instructions using the Uvitec imaging system (Cleaver Scientific).

ChIP seq analysis

Four different ChIP seq datasets (GSM670037; GSM621419; GSM669996; GSM669951) were analyzed using the WashU EpiGenome Browser (http://epigenomegateway.wustl.edu/browser/).

PBMC proliferation assay

Peripheral blood mononuclear cells (PBMCs) were obtained from heparinized blood of healthy donors by conventional Ficoll – Hypaque gradient for 20 min at 600 x g, after obtaining written informed consent. The proliferation of PBMCs in RPMI 1640 medium (ThermoFisher, 11875-093) supplemented with 10% FBS, in response to PHA-P (Sigma-Aldrich, L8754), in presence or absence of MSCs, was evaluated in triplicate in flat-bottom 96-well tissue culture plates. Briefly, MSCs were seeded overnight at MSCs:PBMCs ratio of 1:2, 1:20, and 1:200. The day after, $1x10^5$ PBMCs per well were added with or without PHA-P (4 µg/ml) and pulsed with ^{3}H-thymidine (1µCi/well, specific activity 6.7 µCi/mmole, Perkin Elmer, Waltham, MA) after 48 hours of culture. PBMC proliferation was measured by ^{3}H-thymidine incorporation assay 18 hours later using a Microbeta Trilux 1450 detector. Results were expressed as mean percentage of stimulation index (SI= cpm stimulated/cpm unstimulated).

Flow cytometry

MSC were immunophenotypically characterized by flow-cytometry at passage 2/3 to evaluate the expression of MSC canonical (CD90, CD73, CD105) and primitive (CD146, CD271) surface markers, hematopoietic (CD45, CD34, CD14), endothelial (CD31) and HLA class II surface markers (HLA-DR). Cells were detached and washed with PBS + 2% FBS. 1×10^5 cells were incubated with the proper antibody mix for 10 minutes at RT in the dark. The following antibodies

were used: CD90 PE (BioLegend, 328110), CD105 FITC (BioLegend, 323204), CD73 PE (BD Biosciences 555445), CD146 V450 (BD Biosciences, 562136), CD271 PE (BD Biosciences, 557196), CD45 APC (BD Biosciences, 340910), CD34 FITC (BD Biosciences, 345801), CD14 APC (BD Biosciences, 555399), CD31 FITC (BD Biosciences, 55027) and HLA-DR PB (BioLegend, 307624). Unstained cells were used as negative control. Flow cytometry assay was standardized using rainbows beads (Sperotech, RCP305A). All samples were run on BD FACSCanto II cytometer (BD Biosciences). At least 10.000 were recorded. Analysis of flow cytometry results was performed using FlowJo software (Tree Star Inc.). Frequency was calculated as percentage of positive cells on total cells. dMFI relative to unstained control was calculated as MFI sample – MFI unstained control. ΔdMFI relative to untreated control was calculated as dMFI treated sample – dMFI untreated sample.

The frequency of CD146 positive cells *in vivo* was evaluated in HD- and BT- BM aspirates. After purification of CD34+ from BM MNCs, the CD34 negative fraction was washed twice with PBS + 2% FBS and incubated with the following antibody mix: CD45 APC, CD105 FITC, CD146, 7AAD, for 15 min at RT in the dark. The frequency of CD146 in vivo was calculated as percentage of cells negative for 7AAD and CD45 and positive for CD105 and CD146. All samples were run on MoFlo XDP sorter (Beckman Coulter). Analysis of flow cytometry results was performed using FlowJo software (Tree Star Inc.).

Cell death assay

5 x 10^5 MSCs from healthy donors were plated in basal medium. The day after, iron (40μM) was added to the culture. 5 day later the percentage of death cells was calculated as number of Trypan blue positive cells on total number of cells using a Burker chamber. Experiments were performed in triplicates for each HD samples. Untreated cells cultured for 5 days were used as negative control.

The presence of apoptotic cells after iron treatment was evaluated in MSCs from healthy donors exposed to 40μM iron for 5 days. 5 x 10^5 MSCs were plated in basal medium. The day after 40μM

iron (FAC) was added to the culture. 5 day later the caspase 3/7 activity was measured using the Caspase-Glo® 3/7 Assay (Promega, G8090) according to the manufacturer's instructions. Experiments were performed in triplicates for each HD samples. Untreated cells cultured for 5 days were used as negative control. Results were expressed as relative to Caspase 3/7 activity in untreated controls.

Iron and deferoxamine (DFO) treatment experiments

In specific experiments BT-MSCs were exposed *in vitro* to increasing doses of Ferric Ammonium Citrate (FAC; 5-10-20-40 μM) for 5 to 21 days. In some experiments BT-MSCs were treated *in vitro* with an iron chelating agent (DFO 100μM for 24 h) (1) in the presence or in the absence of FAC.

Luminex Assay

R&D system Luminex Kit was used to analyse cytokine's secretion in supernatant derived from HD- and BT-MSC. Supernatant were collected, filtered with a 0.22μm PVDF filter (Millipore) and stored at -80° or used freshly. Custumized Luminex plates were obtained to screen for: IL1α, IL1β, IL15, MIP-1a. Assays were run as per manufacturers' instructions with standards and samples in duplicate. Data were acquired on a calibrated Bio-Plex MAGPIX multiple reader system (Bio-Rad) and visualized with Bio-Plex manager Software.

Implantation of humanized ossicles into NSG mice.

Humanized ossicles were established according to published protocols (89) (88). In particular, 1 x 10^5 MSCs from 3 different HDs and BT patients were pre-seeded on gelatin scaffold (Gelfoam) in combination with 1 x 10^5 endothelial cells (HUVECs), purified from different cord donors after obtaining written informed consent, and 1 x 10^5 $CD34^+$ cells. Scaffolds were surgically implanted

subcutaneouslly in the flank of non-irradiated NSG mice, under anesthetized with 0,5% isofluorane. All procedures were performed according to protocols approved by the Committee for Animal Care and Use of San Raffaele Scientific Institute. 5 or 12 weeks post implantation humanized scaffolds were explanted from the skin and processed for histological (hematoxylin-eosin staining), flow cytometry (for huCD45 expression) and immunofluorescence (huCD45, hu-vimentin) analysis.

Immunofluorescence

Ossicles were explanted, fixed in 4% PFA and decalcified overnight using EDTA. Ossicle sections were permeabilized with 1% Triton X-100 and processed for immunostaining. The following antibody were used: human CD45 (Dako, M0701, 1:100), human vimentin (LSBio, LS-C204593, 1:200). Nuclei were stained with Hoechst.

References

1. Langlois A, Bietiger W, Mandes K, Maillard E, Belcourt A, Pinget M, et al. Overexpression of vascular endothelial growth factor in vitro using deferoxamine: a new drug to increase islet vascularization during transplantation. *Transplant Proc.* 2008;40(2):473-6.

Supplementary Figures

Suppl. Fig. 1: Expression analysis of iron transporters and ferritin.

A) Expression analysis of *TFR1*, *DMT1*, *ZIP14* and *ZIP8* in HD- and BT-MSCs exposed to increasing dose of iron (5, 10 and 20μM) provided as FAC for 5 days. Results are expressed as fold change compared to untreated samples. B) Representative image of Western Blot analysis of ferritin expression on protein extracts from HD- and BT-MSCs at basal level (ctrl) and after 5 days of iron treatment (40μM) (+iron). C) Representative images of Perl's stianing on MSCs exposed to 40 μM iron for 21 days. D) Expression analyss of *CDKN2A* in HD- and BT-MSCs treated with 40 μM iron for 21 days. Each squared dot represents one HD sample (blue > 18y; light blue <18y). Each rounded dot represents one BT sample (red > 18years; orange <18y). *p*-values were determined by student's *t*-test (ns=not significant).

Suppl. Fig. 2: Expression analysis of iron metabolic genes.

A) Expression analysis of transferrin (*TFR1*) and free iron transporter genes in untreated BT-MSCs (white bars), BT-MSCs exposed to 40 μM iron for 5 days (black stripped bars) and BT-MSCs exposed to 40 μM iron for 5 days and treated 24h with 100 μM DFO (grey stripped bars). Data are expressed as mean ± SEM. Experiments were performed in triplicates; $n \geq 3$; *p*-values were determined by student's *t*-test (*p < 0.05; **p < 0.01; ns = not significant).

Suppl. Fig. 3: Senescence analysis.

A) Analysis of senescence-associated secretory phenotype by ELISA in HD- and BT-MSCs. Each squared light blue dot represents one HD sample. Each rounded orange dot represents one BT sample. *p*-values were determined by student's *t*-test (ns=not significant). Protein amount was normalized for the number of plated cells. **B)** Western blot analysis of total p53 expression on protein extracts isolated from 3 different HD- and BT-MSCs cultured for 5 days in presence of 40

μM iron. Tubulin was used for sample normalization (left panel). Quantification of p53 expression was performed using Image J (right panel).

Suppl. Fig. 4: Analysis of KDM expression and demethylating activity in HD- and BT-MSCs.

A) Expression analysis of histone lysine demethylases (*KDM2A*, *KDM2B*, *KDM3A*, *KDM5A*, *KDM5B*) in HD- (n=7) and BT-MSCs (n=6) by RT-qPCR. Results are expressed as 2-DCT. Each error bars show means ± s.e.m. Each squared dot represents one HD sample (blue > 18y; light blue <18y). Each rounded dot represents one BT sample (red > 18y; orange <18y). *p*-values were determined by student's *t*-test (ns=not significant). **B)** Western blot analysis of total H3, H3K9 3me, H3K36 3me expression on protein extracts isolated from 3 different HD- and BT-MSCs. Tubulin was used for sample normalization.

Suppl. Fig. 5: Analysis of 4 different ChIP sequencing datasets for H3K36 3me on BM derived MSCs.

Example of H3K36 3me analysis using 4 different previously published ChIP seq datasets (GSM670037; GSM621419; GSM669996; GSM669951). H3K36 methylation state of BM niche-associated genes (*CXCL12*, *ANGPT1*, *VEGFA*, *FGF2*, *KITLG*, *IL6*, *CDH2*) and control genes (*GJA1*, *COL2A1*) is represented.

Suppl. Fig. 6: Reduced percentage of HSPCs after *in vitro* co-culture with BT-MSCs.

A) Percentage of different cord blood (CB) HSC subpopulations on CD45 positive cells after 3 days of co-culture with HD-and BT-MSCs in presence or absence of proper cytokines. CB CD34+ cultured for 3 days are used as control. Data are expressed as means ± s.e.m.; n=3**.** **B)** Example of gating strategies used to identify different CB HSC subpopulations after 3 days of co-culture with

HD- and BT-MSCs in presence (+ cytokines) or absence of proper cytokines. CB CD34 cultured alone with or without the addition of cytokines are used as control

Suppl. Fig. 7: Engraftment of human CD45$^+$ cells in NSG mice co-infused with human healthy-donor CB CD34$^+$ and HD- or BT- MSCs.

A) Example of gating strategies used to identify human CD45$^+$ cells in the peripheral blood of mice 6 and 12 weeks after co-infusion of human CB CD34$^+$ with HD- (upper panel) or BT-MSCs (lower panel). **B)** Example of gating strategies used to identify human lymphoid lineages in the peripheral blood of mice 6 and 12 weeks after transplantation. CD19 and CD3 were used to identify B and T cell differentiation.

Suppl. Fig. 8: Histological analysis (H&E) of humanized ossicles explanted 5 weeks post implantation. Representative images of sections derived from decalcified and paraffin embedded humanized ossicles. **A**, **B**) Representative areas of an HD-MSC derived ossicle-section. **C)** Representative area of a BT-MSC derived ossicle-section. Experiments were performed in triplicates; $n \geq 3$. **A**) Black arrows: mature and immature PMNCs; blue arrows: osteoblasts; red arrow: stromal cells; black asterisks: sinusoidal vessels; blue asterisk: bone. **B**) Red arrows: stromal cells; Green arrows: myeloid cells; Black asterisks: sinusoidal vessels; Blue asterisk: bone; green asterisk: immature bone. **C**) Red arrow: stromal cells; blue asterisk: bone.

Suppl. Fig. 9: Altered capacity of BT-dervived ossicles to sustain human hematopoiesis. A) Example of Facs analysis for human CD45 expression on collagenase digested ossicles (left panel). Percentage of human CD45+ cells is reported in the right panel. Experiments were performed in triplicates; $n \geq 3$. *p*-values were determined by student's *t*-test (*$p < 0.05$). **B)** Quantification of

human CD45+ cells expressed as percentage of CD45+ cells on total nuclei. In all panels: white bars = HD-derived ossicles, black bars = BT-derived ossicles. Experiments were performed in triplicates; $n \geq 3$. p-values were determined by student's t-test (*$p < 0.05$). Representative images of HD- (C) and BT-derived (D) ossicle sections **($n \geq 3$)** processed for immunohistochemistry. Vimentin was used to identify human mesenchymal elements. Hematopoietic cells of human origin were identified using anti human CD45.

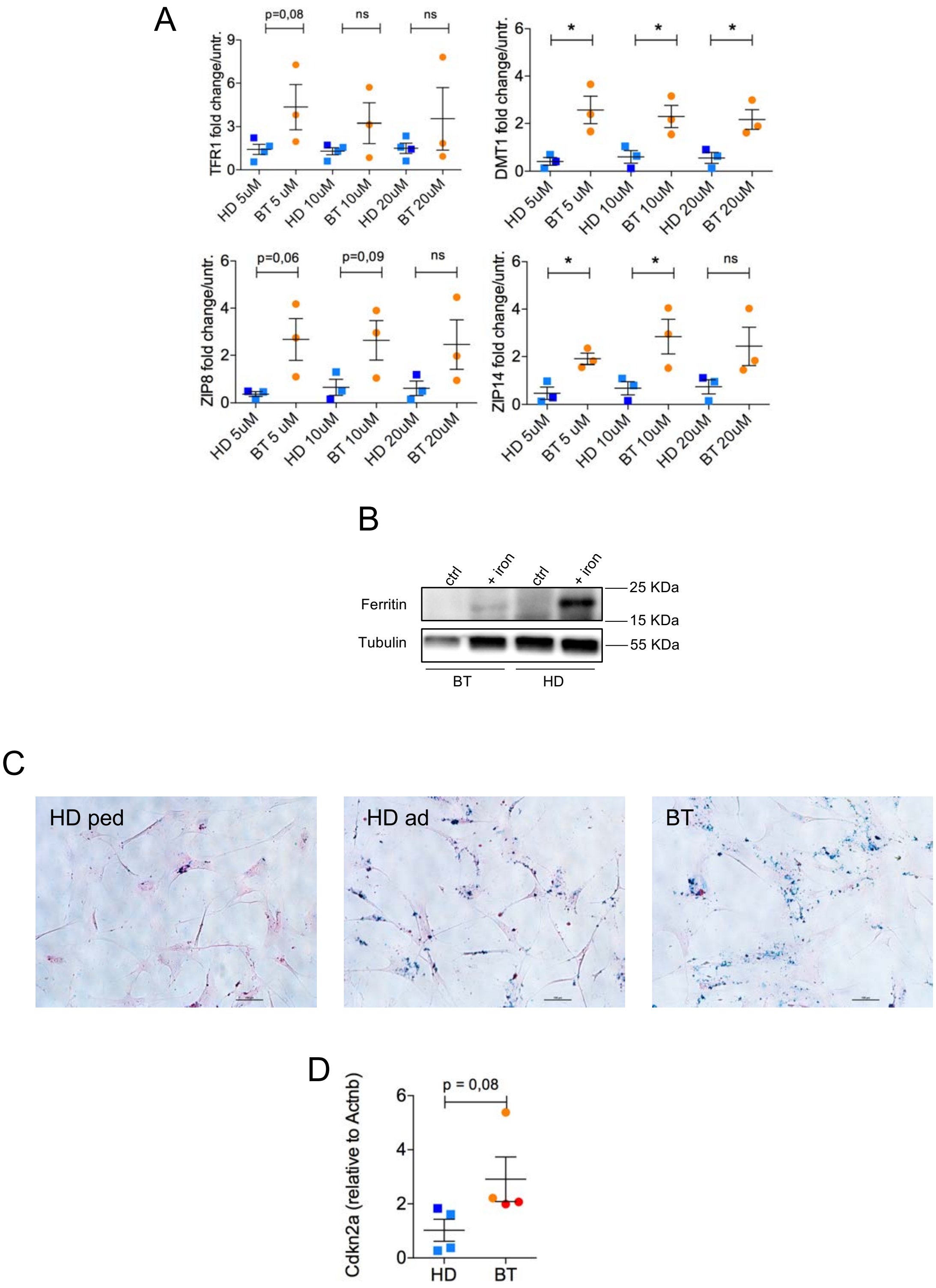

Supplementary Figure 1

A

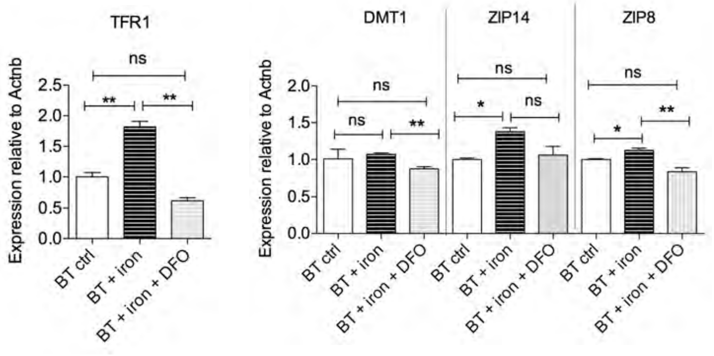

Supplementary Figure 2

A

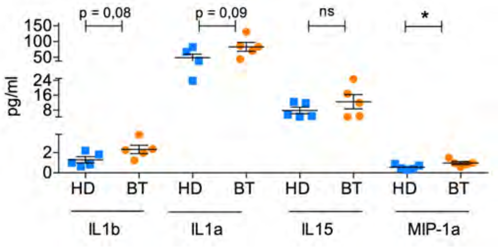

B

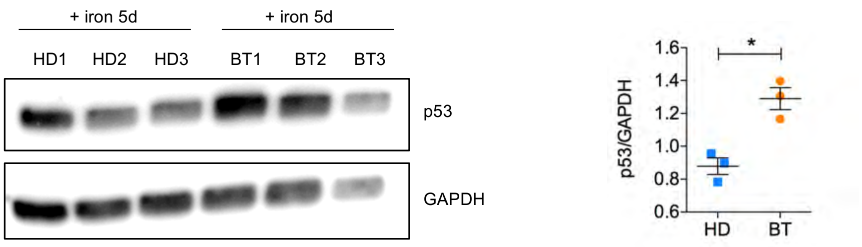

Supplementary Figure 3

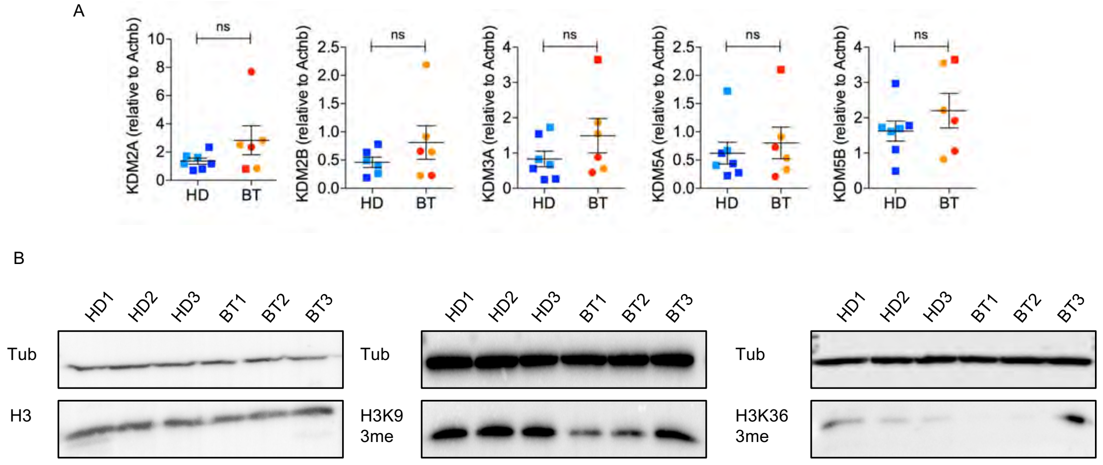

Supplementary Figure 4

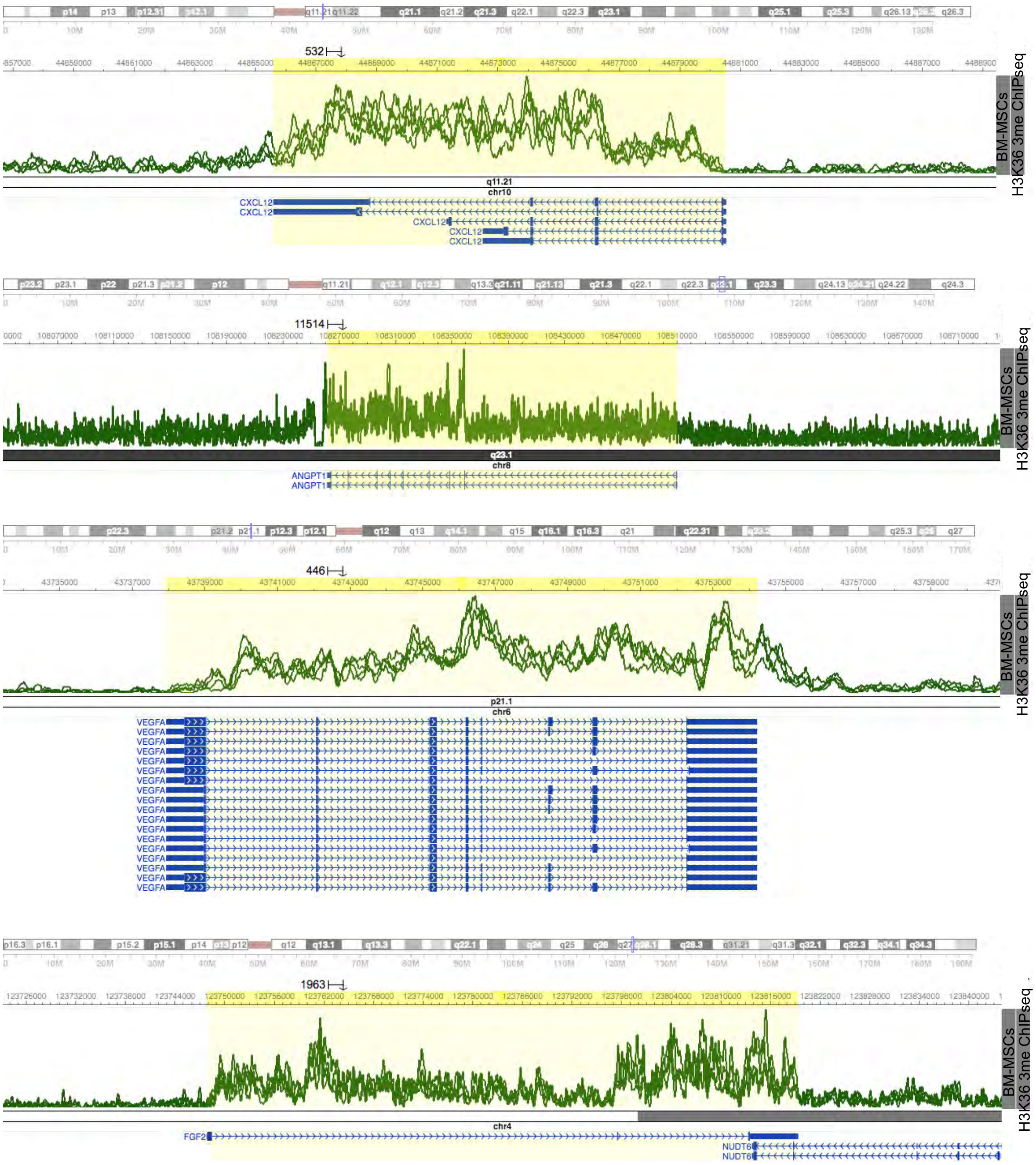

Supplementary Figure 5

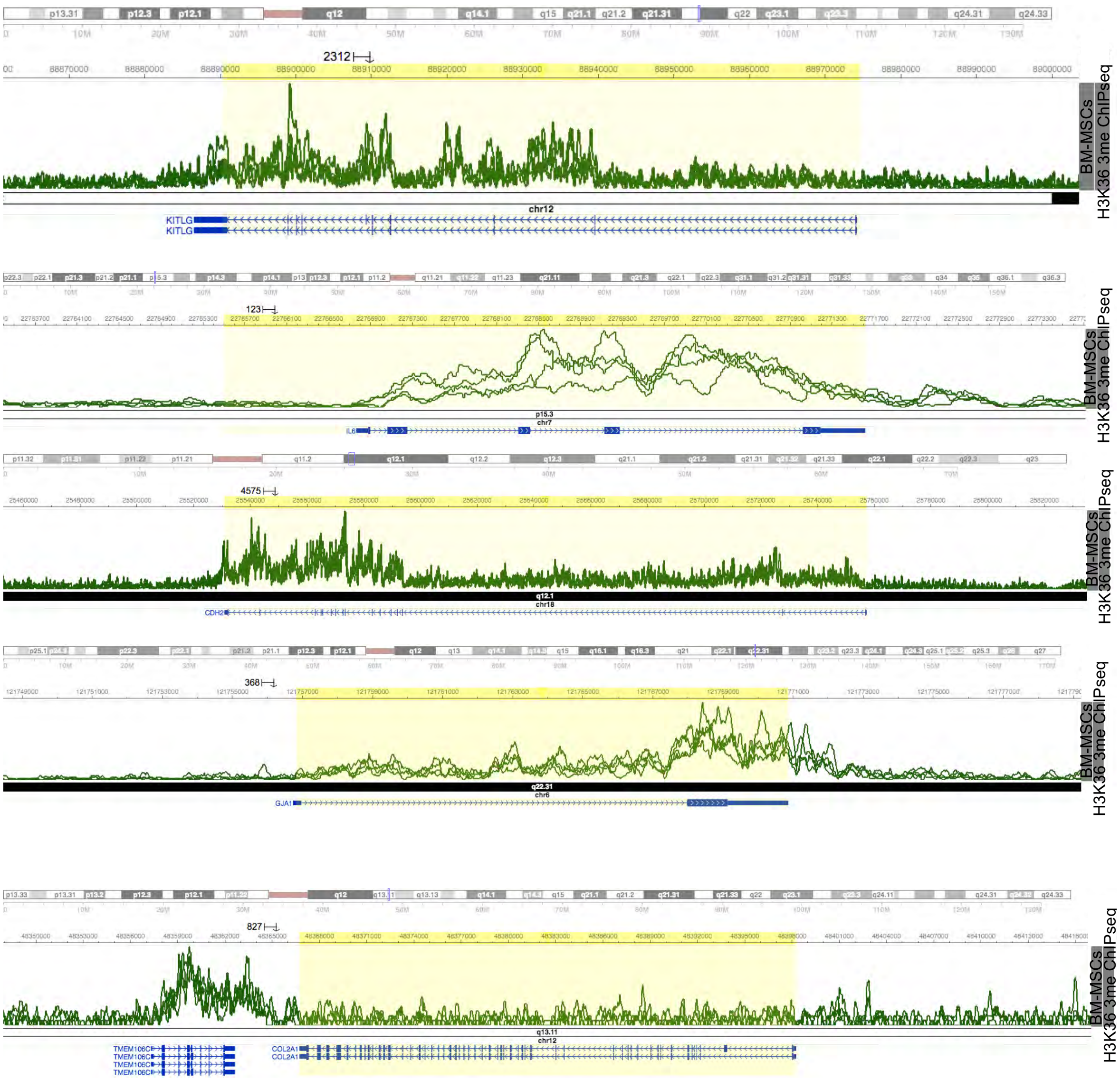

Supplementary Figure 5

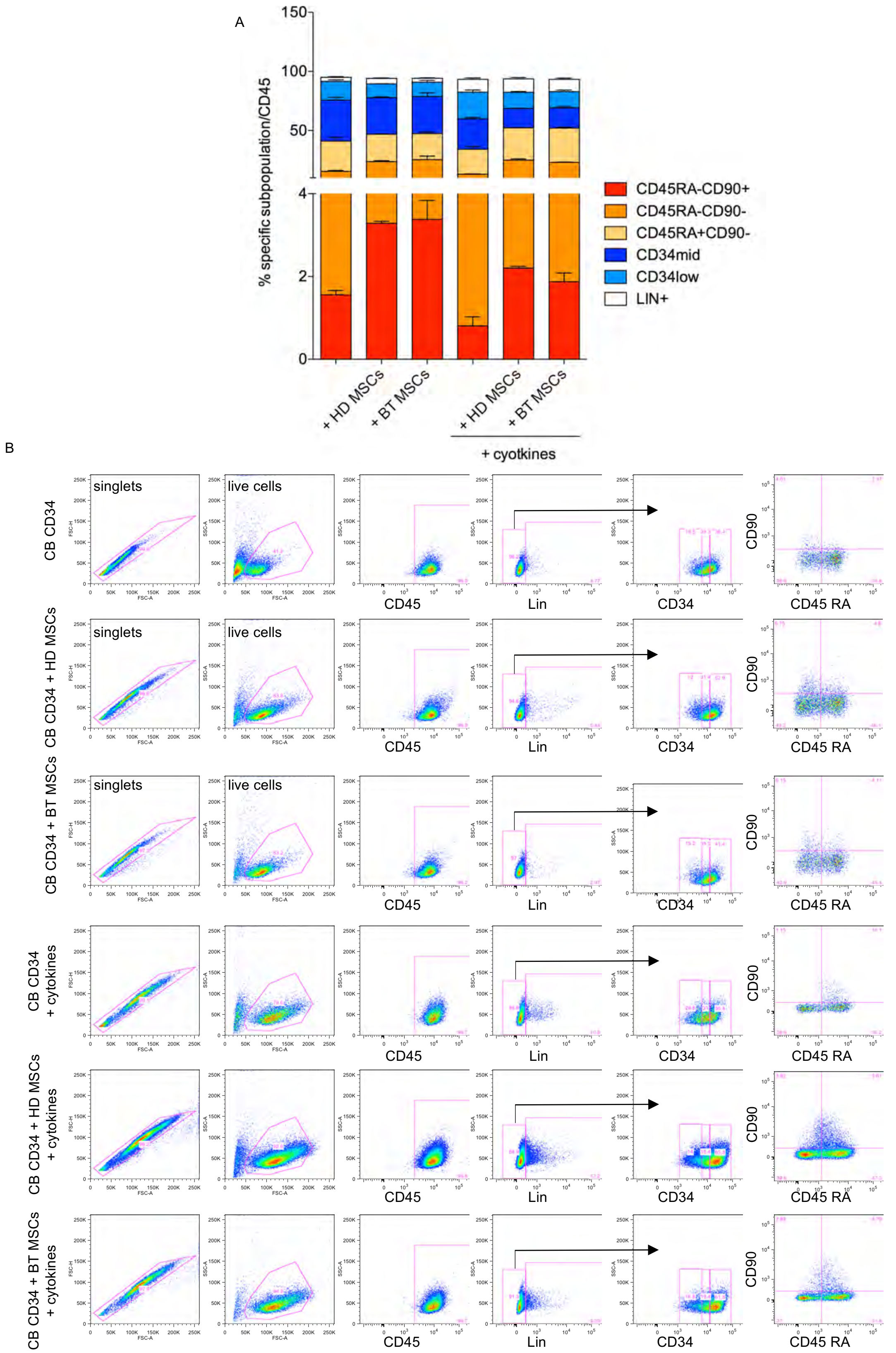

Supplementary Figure 6

A

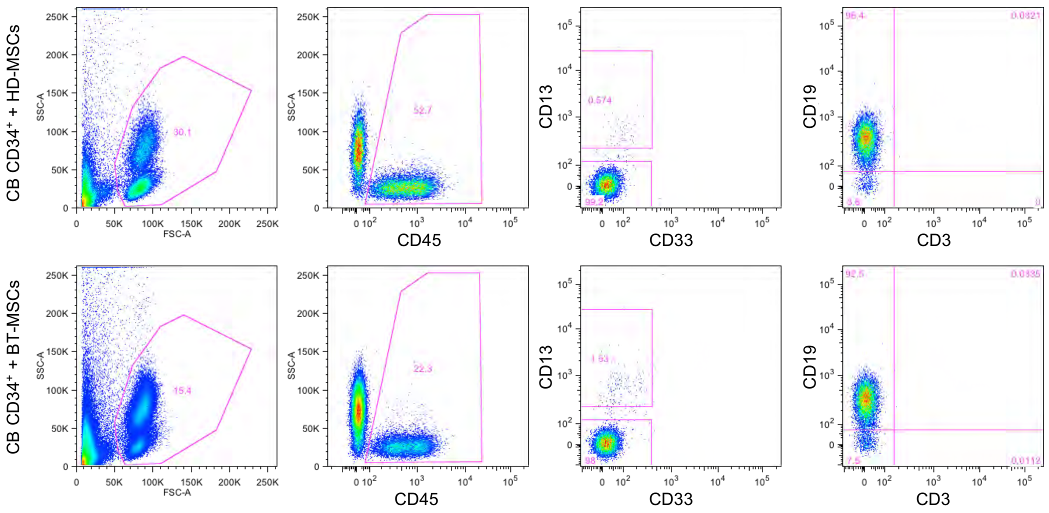

B

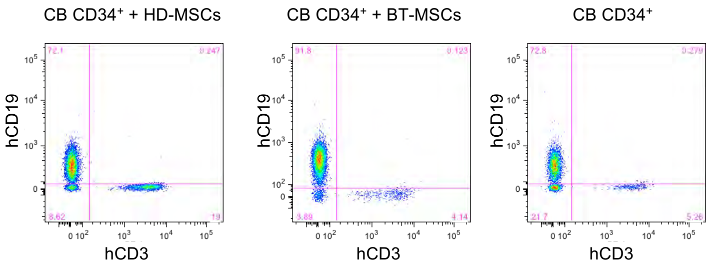

Supplementary Figure 7

A

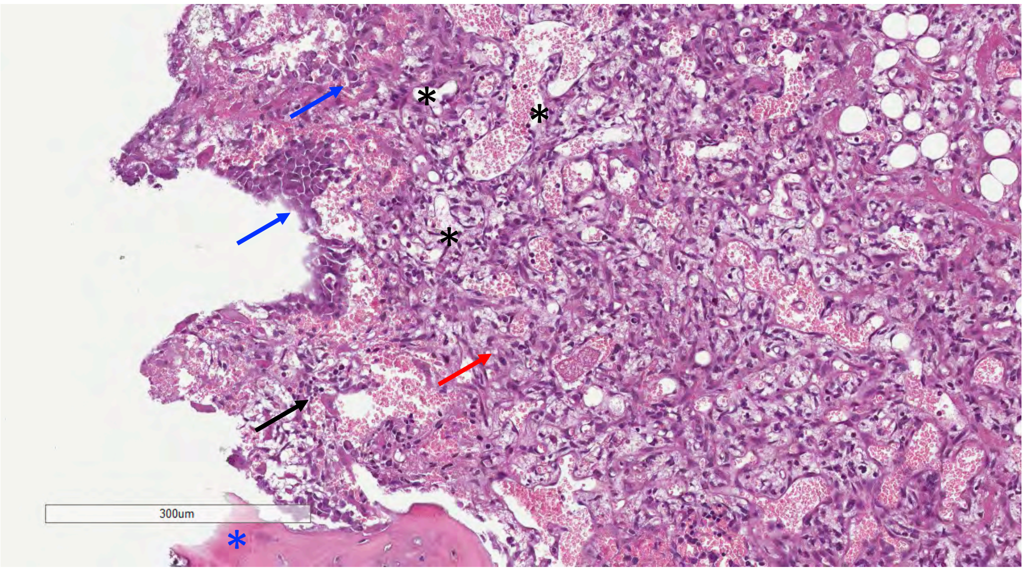

B

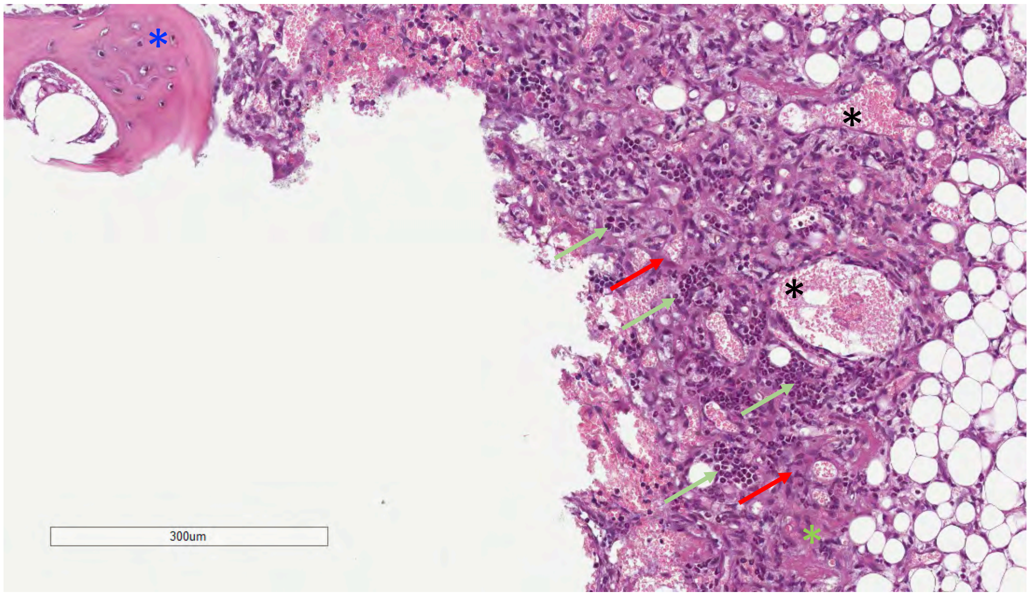

Supplementary Figure 8

c

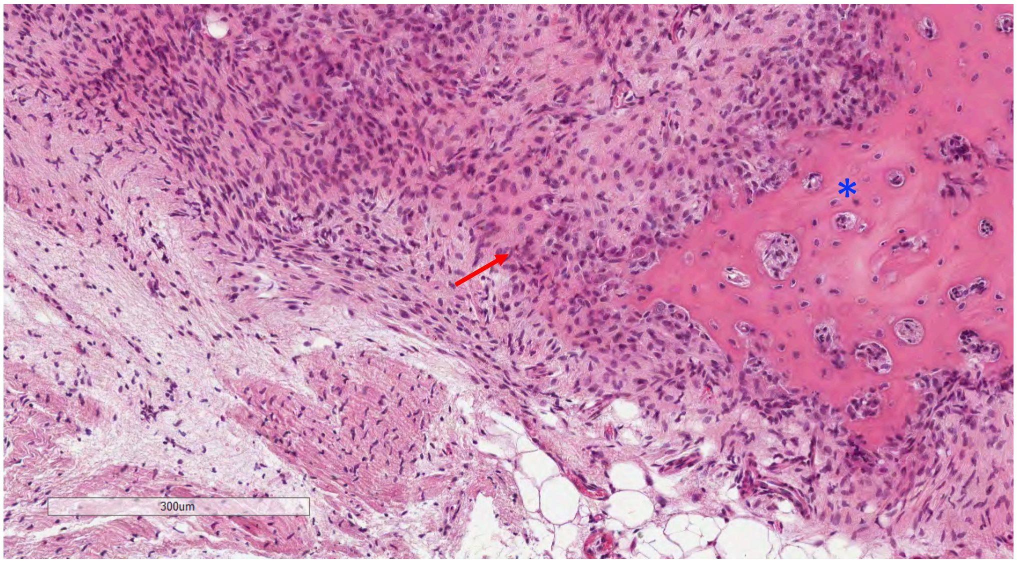

Supplementary Figure 8

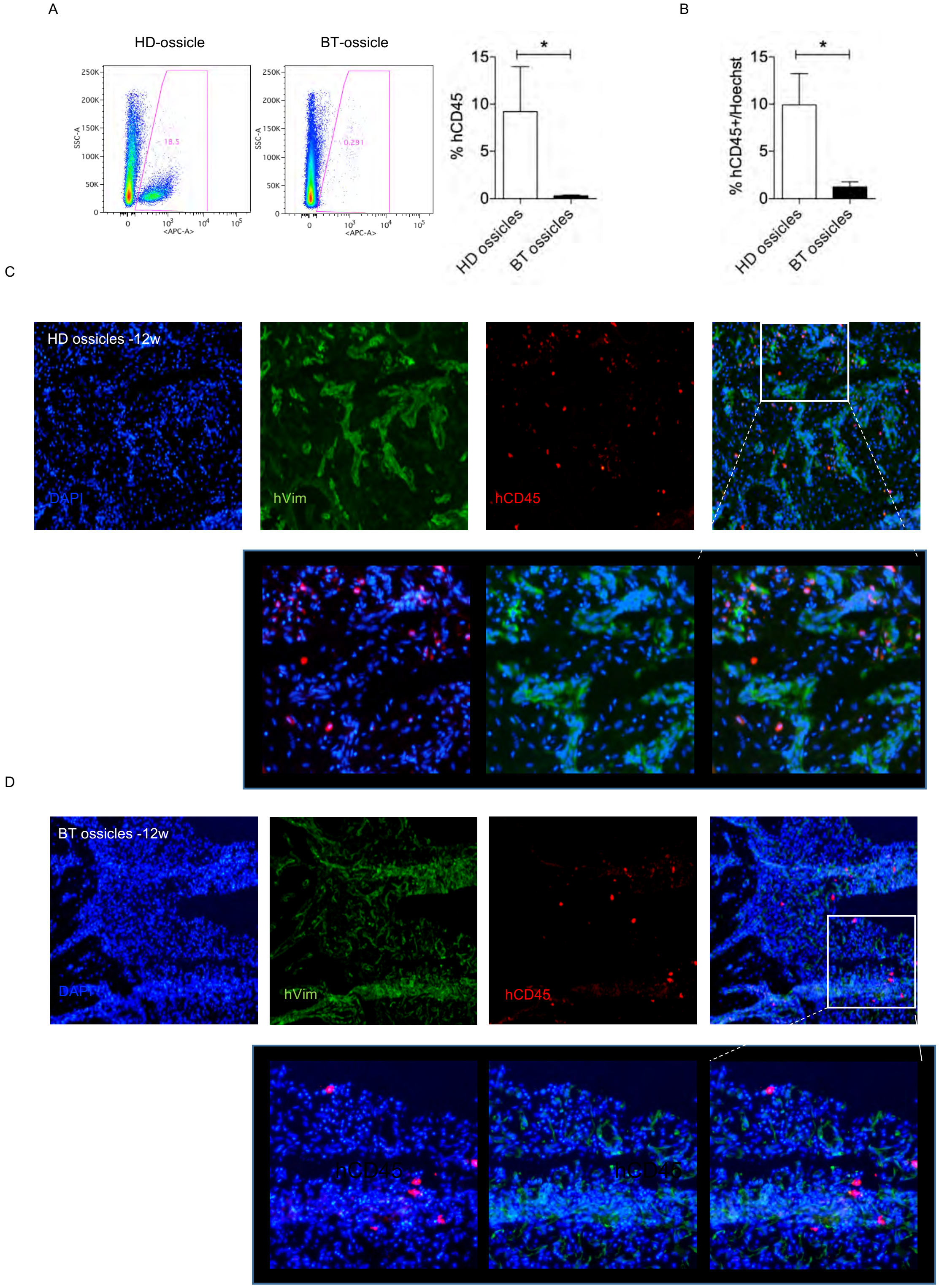

Supplementary Figure 9
